# Experimental rice seed aging under elevated oxygen pressure: Methodology and mechanism

**DOI:** 10.3389/fpls.2022.1050411

**Published:** 2022-12-01

**Authors:** Manjunath Prasad C. T., Jan Kodde, Gerco C. Angenent, Ric C. H. de Vos, Carmen Diez-Simon, Roland Mumm, Fiona R. Hay, Sasiwimon Siricharoen, Devendra K. Yadava, Steven P. C. Groot

**Affiliations:** ^1^ Bioscience, Wageningen Plant Research, Wageningen University and Research, Wageningen, Netherlands; ^2^ Laboratory of Molecular Biology, Wageningen University and Research, Wageningen, Netherlands; ^3^ Department of Seed Science and Technology, ICAR-Indian Agricultural Research Institute, New Delhi, India; ^4^ Department of Agroecology, Aarhus University, Slagelse, Denmark; ^5^ Division of Crop Science, Indian Council of Agricultural Research, New Delhi, India

**Keywords:** EPPO storage, lipid oxidation, *Oryza sativa* L. (rice), seed aging, seed deterioration, seed longevity and viability, seed quality and storage, volatiles

## Abstract

Seed aging during storage results in loss of vigor and germination ability due to the accumulation of damage by oxidation reactions. Experimental aging tests, for instance to study genetic variation, aim to mimic natural aging in a shorter timeframe. As the oxidation rate is increased by elevating the temperature, moisture, and oxygen levels, this study aimed to (1) investigate the effect of experimental rice seed aging by an elevated partial pressure of oxygen (EPPO), (2) elucidate the mechanism of dry-EPPO aging and (3) compare aging under dry-EPPO conditions to aging under traditional moist-controlled deterioration (CD) conditions and to long-term ambient storage. Dry seeds from 20 diverse rice accessions were experimentally aged under EPPO (200 times higher oxygen levels), at 50% relative humidity (RH), along with storage under high-pressure nitrogen gas and ambient conditions as controls. While no decline in germination was observed with ambient storage, there was significant aging of the rice seeds under EPPO storage, with considerable variation in the aging rate among the accessions, with an average decline toward 50% survival obtained after around 21 days in EPPO storage and total loss of germination after 56 days. Storage under high-pressure nitrogen gas resulted in a small but significant decline, by an average of 5% germination after 56 days. In a second experiment, seven rice seed lots were stored under EPPO as compared to a moist-CD test and two different long-term ambient storage conditions, i.e., conditioned warehouse seed storage (CWSS) and traditional rice seed storage (TRSS). Untargeted metabolomics (with identification of lipid and volatile compounds profiles) showed a relatively high increase in levels of oxidized lipids and related volatiles under all four storage conditions. These compounds had a high negative correlation with seed viability, indicating oxidation as a main deteriorating process during seed aging. Correlation analysis indicated that EPPO storage at 50% RH is more related to aging under TRSS at 60% and CD-aging at 75% ERH rather than CWSS at 40% ERH. In conclusion, aging rice seeds under EPPO conditions is a suitable experimental aging method for analyzing variation among seed lots or genotypes for longevity under storage.

## 1 Introduction

Seed longevity or shelf-life, the period over which seeds remain viable during storage, is a vital seed quality trait ensuring the survival of flowering plants from one generation to the next. Long-term survival of seeds in soils, seed (gene) banks, or commercial warehouse storage has wide ecological and socio-economic significance (Li and Pritchard, 2009; Probert et al., 2009). During storage, either in the soil or warehouses, deterioration is inevitable which impairs the germination performance of the seeds (Abdul-Baki and Anderson, 1972). In nature, seeds will drop on the soil surface or are buried, the latter often in relatively moist conditions. For agriculture, seeds are dried and stored under dry conditions to limit deterioration, as humidity is a main environmental factor stimulating seed aging.

The RH during agricultural seed storage can vary depending on the economic value of the seeds. High-value horticultural seeds are usually stored in conditioned warehouses at 30% RH. In contrast, cereal seeds, including farm-saved seeds, are stored in non-conditioned warehouses where ambient humidity and temperature often varies. Commercial rice seeds are often dried to a moisture content of 12%, which is in equilibrium with around 60% RH at 30°C. Subsequent storage in warehouses without humidity control can result in a slow but steady increase in seed moisture, especially during the monsoon season, which can cause a relatively fast decline in seed quality. With an expected increase in the area for direct-seeded rice and the use of more expensive hybrid rice seeds, there is a need for improved seed longevity.

Seeds exhibit considerable variability in seed longevity depending on species, genetics, maternal environment, pre-storage treatments, and storage environment (Walters et al., 2004; Probert et al., 2007; Solberg et al., 2020). For the storage environment, humidity, temperature, and oxygen are the main factors influencing the longevity of the seeds (Roberts, 1961; Roberts and Abdalla, 1968; Roberts and Ellis, 1989). Due to the slow rate of aging reactions in dry seeds, studying symptoms and mechanisms of seed aging under optimal storage conditions takes a long time. Thus, relatively fast (few days up to some weeks) experimental aging tests like the ‘accelerated aging’ (AA) (TeKrony, 1993) or ‘controlled deterioration’ (CD) test (Powell, 1995) are widely used to determine the sensitivity of seeds to deterioration during storage. In these two tests, seeds are exposed to an elevated temperature and moisture content to speed up the aging process. Various studies have used these moist aging methods to determine genetic variation for seed longevity in, for instance, *Arabidopsis* (Bentsink et al., 2000), wheat (Rehman Arif et al., 2012; Rehman Arif and Börner, 2020; Zuo et al., 2020); lettuce (Schwember and Bradford, 2010); oilseed rape (Nagel et al., 2011), barley (Nagel et al., 2009), maize (Revilla et al., 2009) and rice [reviewed in (Hay et al., 2019)]. However, the reliability of such experimental aging tests at humid conditions is questioned by the scientific community since it has become clear that results of moist seed aging tests often show poor correlation with long-term storage under dry conditions (Schwember and Bradford, 2010; Nagel et al., 2015; Agacka-Mołdoch et al., 2016), which also holds true for rice (Hay et al., 2019). One obvious reason could be due to the difference in the physiology of seeds during a moist aging test (at 75% up to 100% RH) and long-term dry storage (at 5 to 35% RH) (Whitehouse et al., 2020).

Under optimum dry and cool storage conditions, as performed in gene banks or with horticultural seeds under controlled warehouse storage, the cytoplasm of seeds is in a glassy state characterized by high viscosity and low molecular mobility (Sun and Leopold, 1994; Murthy et al., 2003; Buitink and Leprince, 2008; Ballesteros et al., 2020). Under moist storage regimes, the seed cytoplasm is in a fluid/liquid state, exhibiting high molecular mobility and allowing a high rate of chemical reactions (Nagel et al., 2019), which is not the case under dry storage conditions with a glassy state cytoplasm. In the glassy state, only spontaneous chemical reactions such as direct (non-enzyme catalyzed) oxidative attack (lipid oxidation), molecular crowding, and the resulting Amadori and Maillard reactions appear to be the cause of molecular damage (Narayana Murthy and Sun, 2000). Moreover, related to the potential of enzyme activity in a liquid cytoplasm, fermentation products (such as acetaldehyde and ethanol) can be produced by seeds aging under moist-warm regimes (Colville et al., 2012; Mira et al., 2016; Gerna et al., 2022).

One of the universal hallmarks of aging is damage to macromolecules (DNA, RNA, and proteins) and bio-membranes caused by reactive oxygen species (ROS) (Benson, 1990; Hendry, 1993; Bailly, 2004; Halliwell and Gutteridge, 2015; Sano et al., 2016). Molecular oxygen is an important source for ROS generation. Early investigations have already pointed to the deleterious effect of oxygen in seeds related to increased chromosomal aberrations and defects (Ehrenberg et al., 1957; Kronstad et al., 1959; Moutschen-Dahmen et al., 1959). However, the effect of oxygen on seed longevity differs depending on whether the seed cytoplasm is in a glassy or fluid/liquid state. Oxygen is relatively more deleterious to seeds in dry conditions (with glassy cytoplasm) compared to those with higher moisture contents (fluid cytoplasm) (Gerna et al., 2022). This also explains the early observations by Roberts (1961) on the effect of oxygen on rice seeds, when compared to air, a positive effect of nitrogen and a negative effect of oxygen storage for seeds at 12% moisture content, but an opposite effect at 14.5% moisture content. In dry seeds, the scavenging of ROS occurs by antioxidant molecules like tocopherols and glutathione rather than enzymatic antioxidants like catalases because these enzymes are not active in a glassy cytoplasm with extreme low molecular mobility (Gerna et al., 2022). Biochemical changes found in wheat or barley seeds stored under AA or CD conditions deviate from those in seeds stored under natural dry or long-term gene bank storage conditions (Galleschi et al., 2002; Roach et al., 2018).

Seeds stored under moist conditions need oxygen for aerobic respiration, but under dry conditions the longevity of seeds can be extended when stored under a gaseous environment devoid of oxygen (Justice and Bass, 1978; Schwember and Bradford, 2011; Groot et al., 2015). Because of the deleterious effect of oxygen under dry storage conditions, Groot et al. (2012) developed an alternative aging assay by storing seeds under dry conditions and normal temperatures, but under a higher oxygen concentration, called ‘EPPO’ which stands for Elevated Partial Pressure of Oxygen. Using lettuce, soybean, and cabbage seeds, they showed that dry aging under EPPO conditions (18 MPa partial pressure of oxygen (P_O2_) at 20°C after equilibration at 35% RH) was physiologically different from moist-warm aging conditions (45°C after equilibration at 85% RH). This included a decline in tocopherol levels under dry EPPO aging that correlated well with the decrease in germinability, while no apparent decrease of this lipophilic antioxidant was observed under moist aging conditions. Subsequently, this EPPO method has successfully been used to study seed aging of barley (Nagel et al., 2016), *Arabidopsis* (Buijs et al., 2020; Renard et al., 2020) and *Allium* sp. (Hourston et al., 2020). Genetic analyses with barley and *Arabidopsis* seeds showed that EPPO storage reveals QTLs for shelf-life that are at least partly different from those identified under more humid aging conditions (Nagel et al., 2016; Buijs et al., 2020; Renard et al., 2020). Moreover, EPPO aging with *Arabidopsis* seeds mimicked after-ripening (dormancy release by dry storage) and dry laboratory bench aging better than the more humid CD assay (Buijs et al., 2018; Buijs et al., 2020).

The overall objective of this study was to test whether EPPO can be applied as an experimental method to study rice seed aging under dry storage conditions. We compared seed longevity parameters and metabolite profiles of seeds aged under the dry-EPPO method to those exposed to a CD test, as well as conditions mimicking conditioned warehouse seed storage (CWSS) and following traditional rice seed storage (TRSS) practices. When applicable to rice, such a method has the potential to decipher the genetic architecture underlying rice seed longevity under dry conditions and to select lines suitable for future breeding purposes.

## 2 Materials and methods

### 2.1 Seed material

The study included two sets of experimental seed samples: I) 20 different rice seed lots of different accessions were used in an experiment to study their aging under EPPO conditions, and II) seven other seed lots to compare their aging under different storage conditions (**Supplementary Table 1**). For the first experiment, the 20 seed lots were provided by the T.T. Chang Genetic Resources Center, International Rice Research Institute (IRRI), Philippines. These seeds were produced during the 2013 dry season, dried in the gene bank drying room (15% RH and 15°C) over 14 days, and then stored at 2-4°C in sealed laminated aluminum foil packets (Whitehouse et al., 2015). For the second experiment, comparing EPPO aging with natural aging and CD conditions, seven seed samples were taken from commercial seed lots produced in India during the wet season of 2014 and 2015. The seeds of varieties Thanu, IR64, and Raksha were obtained from the Zonal Agricultural Research Station (ZARS), Mandya, India, while those of varieties PB 2511 and PB 1509 were obtained from the Seed Production Unit, ICAR-Indian Agricultural Research Institute, New Delhi, India. Seed lots produced in 2014 were stored for seven months in a seed warehouse in India without any control for temperature and RH. In contrast to the usual procedure, the seed samples used in this study neither received a hot water treatment nor chemical treatments for eliminating potential nematodes or fungal pathogens because such treatments may interfere with the seed aging assays. Upon arrival in the laboratory at Wageningen University & Research (WUR), The Netherlands, the water activity of seed samples was determined (see below). Seeds were equilibrated for 14 days in a dedicated storage cabinet with circulating air maintained at 30% RH and 20°C. After this equilibration, seed samples were hermetically sealed inside laminated aluminum foil packets and stored at 20°C until use in the various experiments. A small quantity of seeds from each seed lot were also stored at both -28°C and -80°C for later use.

### 2.2 Seed water activity and equilibrium relative humidity measurements

Water activity (a_w_) of at least 100 seeds per sample was measured at approx. 20°C using an HC2-AW probe connected to a HygroLab 3 display unit (Rotronic Measurement Solutions, Switzerland). The equilibrium relative humidity (ERH) is the RH of the air surrounding the seed that is in equilibrium with its environment. When the equilibrium is achieved, the ERH (%) was subsequently calculated by multiplying the a_w_ by 100 (Kong and Singh, 2016).

### 2.3 Measuring oxygen levels in the seed storage jars

Oxygen levels were measured using an optical oxygen sensor spot (Pst3 type, PreSens Precision Sensing GmbH, Germany, https://www.presens.de), placed against the inner wall of transparent glass storage jars, in conjunction with a fiber optic oxygen meter (PreSens Precision Sensing GmbH). Oxygen readings were recorded at room temperature (RT; approx. 20°C). Before measurement, the oxygen meter was calibrated using an oxygen sensor spot placed inside a jar filled with a sodium azide solution (0% oxygen) and with fresh air (21% oxygen). The PreSens oxygen measurement showed slight fluctuations in the reading (< 1%), hence the lowest of three sequential readings was recorded as the actual oxygen level.

### 2.4 Seed aging treatments

#### 2.4.1 Aging under EPPO conditions

Experimental aging of rice seeds under EPPO conditions was carried out as described by Groot et al. (2012) with slight modifications. Rice seeds with an a_w_ of 0.40 were obtained by equilibrating at 40% RH at 20°C in a controlled humidity cabinet for 18 days (final moisture content, MC, of 9.98%). About 5g of equilibrated seeds from each seed lot were put in a perforated polystyrene tube. The tubes were placed along with ~200g silica gel, equilibrated at the same RH as the seeds, inside a 1.5-liter steel tank, which was closed immediately. The main purpose of including the equilibrated silica gel was to buffer the RH inside the tank upon filling it with dry gas. The steel tanks were then filled slowly (at approximately 0.6 MPa per minute) with compressed air until the pressure inside the tanks reached 20 MPa (200 bar). This resulted in a partial oxygen pressure (P_O2_) of 4.2 MPa, called EPPO treatment (**Supplementary Table 2**). Filling of tanks was done by placing the tanks inside a tub filled with chilled water to avoid heat build-up during filling. After completing the filling process, the tanks were left on the laboratory bench (20°C) overnight before checking the gas pressure. The control treatment for the elevated pressure in EPPO tanks was accomplished by filling the tanks until 20 MPa with nitrogen gas (Grade, Nitrogen 5.0; Purity, ≥99.999%; Linde Gas, Schiedam, The Netherlands), called elevated partial pressure of nitrogen (EPPN) treatment. In the EPPN treatment, the P_O2_ was still 0.021 MPa, due to the air naturally present in the tank before adding compressed nitrogen gas. Due to the high pressure, a theoretical increase of the a_w_ is expected (Okos et al., 2019). According to this theory, the 20 MPa pressure in the tanks is estimated to increase the a_w_ of the seed samples from 0.40 to 0.46, i.e., from 40% RH to 46% RH. Another control treatment was performed by placing the seed samples and 40% RH equilibrated silica gel inside a 1.5-liter Kilner glass jar at ambient pressure (0.1 MPa). The P_O2_ in this ambient pressure control treatment was also 0.021 MPa. The pressurized EPPO or EPPN tanks and the ambient pressure jars were placed in an incubator at 35°C for different durations. Transferring the hermetically closed glass jars from 20°C to 35°C resulted in an increase of the ERH from 40 to 43.5%, as measured with inserted data loggers (EL-USB, Lascar electronics). Though these data loggers could not provide an accurate reading under 20 MPa pressure, combining the measured RH of 43.5% in the glass jars with the theoretical effect of the elevated pressure, it was estimated that the ERH during EPPO or EPPN storage is around 50%. The samples for determining initial quality, i.e., zero days of storage (DOS), were sealed in laminated aluminum foil packets and placed at -28°C until used for germination testing.

At each sampling time, the tank(s) and jars were held on the laboratory bench to reach RT (approx. 20°C) and checked for any leakage during storage by measuring the pressure inside the tank. Then the pressure in the tank(s) was slowly released with an average relative pressure decline at a maximum of 0.5% per minute, using a home-built computerized flow control device. After releasing the pressure, the seed samples were retrieved and the a_w_ of the seeds was measured. Seed samples were subsequently transferred into paper bags (107 mm × 63 mm, L × W) and dried inside a cabinet maintained at 30% RH and 20°C overnight, after which the germination tests were carried out. Subsamples for metabolite analysis were hermetically sealed and stored at -28°C until analyses.

We observed differences in germination values for some seed lots between the two control treatments (21 days ambient control and 21 days EPPN pressure control). Therefore, replication-wise, we corrected all germination trait values in the EPPO aging treatment for the difference observed between the ambient control and the pressure control in each seed lot/accession. These corrected EPPO values are depicted as ‘ΔEPPO’.

#### 2.4.2 Aging under Controlled Deterioration (CD) conditions

Rice seeds with an a_w_ of 0.75 were used for experimental aging by CD. About 5g of seeds from each seed lot were sealed inside perforated plastic bread storage bags (60 mm × 50 mm; L × W). Seeds were equilibrated in a cabinet set to 75% RH at 20°C for 16 days. Subsequently, the seed bags were hermetically packed in laminated aluminum foil packets containing individual samples of seven seed lots and incubated at 35°C. At the end of each storage time, the seed samples were transferred into paper bags and dried overnight at 30% RH and 20°C. The germination test was conducted the next day. Subsamples for metabolomics were hermetically sealed and stored at -28°C until analysis.

#### 2.4.3 Aging under conditioned warehouse seed storage (CWSS) conditions

To mimic storage conditions recommended for medium-term seed storage, seeds were stored at 28°C after equilibration at 20°C and 40% RH for 14 days in a cabinet with circulating air. The final moisture content of these seeds was 9.41% (fresh weight basis). About 5g of seeds from each seed lot were sealed in perforated plastic bread bags. In this experiment, transparent glass jars (194 mL) with a metal lid with plastisol lining were used to store individual seed samples together with buffering silica gel equilibrated at 40% RH. An oxygen sensor spot was attached to the inner side of the glass jar. All the seed storage jars were flushed with air for two minutes at a flow rate of 500 mL min^-1^ through a hole (3 mm diameter) in the metal lid. After flushing, the hole was immediately closed with adhesive aluminum tape (Griffon, the Netherlands) to make the jars airtight. The jars were left on the laboratory bench for four hours before recording the oxygen readings. Except for initial control samples (0 DOS), all jars were transferred to an incubator maintained at 28°C. The control seed samples (0 DOS) were hermetically sealed inside laminated aluminum foil packets and stored at -28°C until used for germination testing. For each storage period, the jars were taken out from the incubator and kept at RT (20°C) for at least three hours before oxygen in each jar was recorded and the a_w_ of the seed samples measured. The seed samples were then transferred into paper bags and placed at 30% RH at 20°C overnight before germination testing. Subsamples for lipid and volatile analyses were hermetically sealed and stored at -28°C.

#### 2.4.4 Aging under traditional rice seed storage (TRSS) conditions

To mimic conditions recommended for short-term seed storage, often used in current practice, herewith referred to as TRSS, the seeds were equilibrated at 20°C and 60% RH, and stored in glass jars at 28°C (resulting in 12.2% moisture content on a fresh weight basis). The experimental setup was otherwise the same as described above for CWSS.

### 2.5 Seed germination assay

Seed germination assays were performed as described in the *International Rules for Seed Testing* (ISTA, 2018) with slight modifications. Two replications of 40-45 seeds per treatment were used in a randomized complete block design. Dry seeds were sown on two layers of dry filter paper (142 mm × 203 mm (L × W) blue blotter paper; All Paper, Zevenaar, the Netherlands) placed in plastic trays (150 mm × 210 mm (L × W); DBP Plastics, Antwerpen, Belgium). Germination was initiated by dispensing 50 mL of demineralized water to each tray. Watered trays were stacked along with one empty watered tray, each at the bottom and on the top. The stack of watered germination trays (maximum of 18) was wrapped in a transparent plastic bag to prevent moisture loss due to evaporation. The prepared stacks were placed in an incubator or climate chamber maintained at 25°C and continuous dark conditions. Germination was followed daily for up to 14 days by making images at frequent intervals using a digital camera (Nikon D80, http://www.nikon.com). Seeds were considered germinated if the radicle protruded by at least 2mm. Germination parameters, namely, total seed germination on the 14^th^ day (G_MAX_, in percentage) and time for 50% germination (t_50_, in hours) were calculated by automatically scoring the germination over time with GERMINATOR software (Joosen et al., 2010). Seedlings with an intact and healthy shoot and root system were evaluated as normal seedlings (**Supplementary Figure 1**) as described in the *ISTA Handbook on Seedling Evaluation* (Don, 2006). Total normal seedlings (TNS; percentage) were determined on the 14^th^ day of the germination test.

### 2.6 Metabolomics analysis

To check whether the different aging treatments affected the metabolism of the rice seeds, metabolomic analysis of the lipid fraction and the volatile compounds was carried out, as detailed below.

#### 2.6.1 Lipidomics

Dehulled seed samples were finely ground in liquid nitrogen. Lipid-soluble compounds were extracted as previously described (Remmers et al., 2018). Fine powder of seed material (10mg) was mixed with 1800µl of cold chloroform/methanol (1:1, v/v) with 0.6% butylated hydroxytoluene (BHT; antioxidant) and 10 µM of 1,2-didecanoyl-sn-glycero-3-phosphocholine (Sigma P7081) as an internal standard. The mix was vortexed, cooled on ice for 20 min, and sonicated for 20 min (2 cycles each) before centrifugation at 20,000 rpm at ambient temperature for 15 min. The supernatant (1200µl) was transferred to a new Eppendorf tube, and the chloroform phase was evaporated using a SpeedVac (Thermo Fisher Scientific, Breda, the Netherlands). The lipid-soluble compounds were re-dissolved in 200µl ethanol (96%), vortexed and sonicated for 5 min, followed by centrifugation at 20,000 rpm for 10 min. Lipidomic analysis was performed by high-performance liquid chromatography (LC) coupled to high-resolution mass spectrometry (LC-MS), as described in Remmers et al. (2018). An LTQ-Orbitrap XL hybrid FTMS (Thermo Fisher Scientific, Breda, The Netherlands) detected compounds eluting from the HPLC column at a mass resolution of 60,000 (FWHM), in positive electrospray ionization mode and at a mass range of m/z 112-1400. The two most contrasting seed samples, i.e., initial and final storage time points of the EPPO treatment, were selected for extraction and analysis in triplicate, referred to as technical quality control (QC) samples. These samples represent the overall analytical variability, including variation due to extraction, LCMS analysis and data processing.

#### 2.6.2 Headspace volatile analysis

Volatile compounds were analyzed by headspace solid-phase microextraction gas chromatography mass spectrometry [HS-SPME-GC-MS] following the procedure described by Diez-Simon et al. (2020). Briefly, 300 mg of ground seed powder was weighed into clean 10mL glass vials and immediately closed with PTFE coated silicon septa screw cap (Supelco, PA, USA). Each sample vial was incubated at 50°C for 10 min with agitation at 250 rpm directly before extraction. Volatile compounds were extracted from the headspace by exposing them to an SPME fiber, after inserting through the septum of the glass vials at 50°C for 20 min without agitation. The fiber was coated with Polydimethylsiloxane/Divinylbenzene/Carboxen (PDMS/DVB/CAR) 50/30 µm diameter, 10 mm length (Supelco, PA, USA). Volatiles absorbed by the SPME fiber was then thermally desorbed in the GC injection port at 250°C for 2 min with a constant helium flow of 1mL/min onto a GC column in splitless mode. Trapping and injection were fully automated using a multipurpose sampling robot (MPS-2, Gerstel, Mülheim, Germany) and operated using Gerstel MAESTRO software version 3.2. GC-MS analysis was performed on an Agilent GC7890A coupled to a 5975C quadrupole mass spectrometer (Agilent Technologies, USA). The complex mixture was separated on Zebron ZB-5MS Plus column (30 m × 0.25 mm i.d. × 1.00 µm film thickness, Phenomenex). The GC oven temperature was programmed as follows: initiated at 45°C for 2 min, then increased at 5°C/min to 250°C and held for 5 min. The column effluent was ionized electron impact at 70eV, and the mass spectra were obtained with a scan range m/z 33-330. The MS interface temperature was set to 280°C. Blank controls and stock seeds of a different rice seed lot of same cultivar IR64-21 were included as quality control (QC) samples because of limited quantity of experimental seed samples remaining after conducting germination to assess analytical variation due to sample extraction, GCMS analysis and data processing.

#### 2.6.3 Metabolomics data processing

Raw LC-MS and GC-MS data files were processed using an untargeted metabolomics workflow centered around the software tools metAlign (Lommen, 2009) and MSClust (Tikunov et al., 2012) as described before Remmers et al. (2018); Diez-Simon et al. (2020). This workflow includes mass peak picking, alignment, filtering, and regrouping of mass features into compounds.

For the LC-MS data, this resulted in the relative intensity values and extracted ion-source mass spectra of over 652 lipid-soluble compounds for each sample. Finally, all intensity data were corrected for variation in the internal standard before further statistical analysis. Selected lipid compounds were annotated by matching the observed accurate *m/z* (after correction) of the H^+^ and NH4^+^ adducts with available lipid databases, including LIPID MAPS (https://www.lipidmaps.org/) and oxidized lipid species reported in aged wheat seeds by Riewe et al. (2017), allowing a mass deviation of maximum 3ppm. All compounds with no or a lower level of identification were considered “Unknowns”.

Processing of the GC-MS data resulted in the relative intensity data for 224 volatile organic compounds. Volatile compounds were identified by matching the mass spectra and retention indices to authentic reference standards or commercial and in-house libraries (NIST17 Mass Spectra Library v.2.3). Retention indices were calculated based on a series of alkanes (C8-C22) which were injected using the same settings as for the samples.

### 2.7 Statistical analysis and data visualization

The germination parameters used in this study were calculated by automatic scoring and curve fitting germination data in time (until 336hrs) for each genotype-treatment combination using the GERMINATOR package (Joosen et al., 2010). Germination parameters were calculated as the mean of the two replicates in the germination test. Descriptive statistics, statistical analysis, principal component analysis (PCA), and graphing were performed in OriginPro 2022 (https://www.originlab.com). Three-way analysis of variance (ANOVA) was applied to observe storage time-aging treatment, genotype-aging treatment-storage time and its interaction effects on seed germination parameters. Paired t-tests were used to compare the means of aging treatments at different storage time points. Pairwise Pearson’s correlation coefficient was estimated to compare seed germination and longevity (*P*_50_; see below) under different storage conditions. For all experiments, significance was determined as *p*<0.05 (*p<0.05, **p<0.01, ***p<0.001). Box plots represent standard box setting with first and third quartile split by the median and the whiskers extend to a maximum of 1.5× interquartile range beyond the box.

Probit analysis of seed survival data was performed using GenStat (19^th^ Edition, VSN International Ltd, United Kingdom, https://www.vsni.co.uk/software/genstat), to fit the seed viability equation (1) (Ellis and Roberts, 1980) and estimate seed longevity:


(1) $$v=K_{\text{i}}-\left(p/\sigma \right)$$

Where *v* is the probit proportion of viable seeds in a seed lot stored for a period of *p* days, *K*_i_ is the initial probit viability and σ is the length of time (days) for viability to fall by 1 probit. The *P*_50_ value, indicating the storage period required for viability to be reduced to 50%, was calculated by modeling the survival data according to Whitehouse et al. (2015). The higher the *P*_50_ value, the more tolerant the seeds are to aging treatment.

For those seed lots/accessions exhibiting dormancy, the breaking of dormancy is described by the equation:


(2) nd=Knd+pσnd

in which *nd* is the probit proportion of non-dormant seeds in the seed lot after *p* days in experimental storage, *K*_nd_ is the fitted initial probit proportion of non-dormant seeds and σ_nd_ is the time it takes for the proportion of dormant seeds to reduce by 1 probit. Equations (1) and (2), were fitted simultaneously as a combined model describing the final observed germination (product of the proportions of non-dormant and viable seeds; Kebreab and Murdoch, 1999).

## 3 Results

### 3.1 Effect of high-pressure oxygen on seed germination and seedling quality

**Figure 1:**
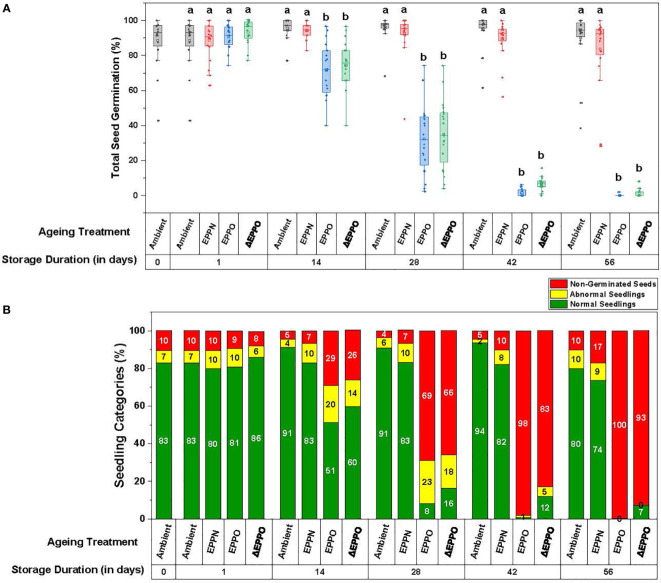
Phenotypic variation in seed germination parameters after storage. **(A)** Total seed germination as the mean for the 20 rice accessions. Different letters above the bars in represent statistically significant differences (*p*<0.05) using Tukey’s test. **(B)** Seedling categories as the mean for the 20 rice accessions.

To establish an EPPO aging protocol for dry rice seeds, seeds of 20 different accessions were stored in a pilot experiment under ambient, EPPN, and EPPO conditions at 35°C for different storage durations. Under the control ambient pressure storage condition, a small aging effect was observed among the individual accessions, indicated by a decrease in total germination and normal seedlings and an increase in the germination rate (t_50_) (**Figure 1** and **Supplementary Table 3**). After 56 DOS, seven lots showed a significant decline in total germination and an apparent delay in the rate of germination. Five of these lots also showed a significant decline in total normal seedlings. One seed lot (accession IRGC 117269) showed only 50% of normal seedlings after 56 DOS. Seed lots from three accessions (IRGC 117266, IRGC 117267, and IRGC 117283) initially showed some dormancy and after-ripening occurred during storage since their maximum germination values initially increased during the first 14 days, to remain at a high level for the subsequent days under ambient storage.

Under the pressure control (EPPN) storage condition, the average seed germination after 56 days (83%) was not significantly different from the ambient control (90%) (**Figure 1A** and **Supplementary Table 3A**). However, variation was observed in the response of individual seed lots. Out of the 20 seed lots analyzed, 10 showed a decline in maximum germination after 56 days storage under the EPPN treatment. These 10 included the seven seed lots that also showed a decline in total germination under ambient control storage. The seed lots of accessions IRGC 117273 and IRGC 117282 showed a specific sensitivity to the high-pressure storage, as their maximum germination was reduced after both 42 and 56 days under EPPN storage, while this reduction was not observed under ambient control storage. For 17 out of the 20 seed lots, storage under EPPN condition for 56 days resulted in a longer delay in seed germination rate (t_50_) compared to the ambient control (**Supplementary Table 3C**). The three seed lots that showed dormancy and after-ripening under ambient storage showed the same under EPPN storage.

The EPPO treatment induced relatively faster deterioration of the seeds (**Figure 1** and **Supplementary Table 3**). Prolonged storage under EPPO resulted in a clear decline in average total germination, average normal seedlings, and delay in germination (higher t_50_ values). Four seed lots (IRGC 117270, IRGC 117274, IRGC 117279, and IRGC 117282) were clearly more sensitive to EPPO storage than the others, as the maximum germination of these lots had declined below 10% after 28 days storage (**Supplementary Table 3A**). The average germination in three of these four seed lots declined relatively quickly under ambient control storage and for all four under EPPN storage. Two accessions (IRGC 117267 and IRGC 117283) were relatively more tolerant to the EPPO treatment, with more than 50% germinating seeds after 28 days. A similar pattern was observed for the percentage of normal seedlings (**Supplementary Table 3B**). While after the longest period (56 days) of storage under ambient or EPPN conditions, 80% or 74% of the total seeds, respectively, still developed into normal seedlings, under EPPO storage, this percentage rapidly declined from 51% at 14 DOS to 8% at 28 DOS and 0% at 56 DOS (**Figure 1B**). After 14 DOS, the seeds took longer to germinate (average t_50_ of 113.6 h) when compared to the ambient control (63.5 h) and EPPN pressure control storage (69.5 h) (**Supplementary Table 3C**).

As the pressurized air used in the EPPO treatment is 78% nitrogen gas and a negative effect of EPPN, compared with the ambient control, was observed for a few seed lots, the germination data obtained under EPPO treatment were corrected for the seed lot-specific high-pressure sensitivity, depicted as ΔEPPO (**Supplementary Table 3**). These pressure-corrected ΔEPPO values are already significantly different from the control treatments at 14 DOS for all the seed germination and seed quality parameters measured, indicating a specific and negative effect of elevated oxygen levels during seed storage.

The different seed germination and seedling quality parameters were highly correlated with each other, and the PCA clearly separated the results of the EPPO aging treatment and its corrected values (ΔEPPO) from the control treatments after 28 DOS (**Supplementary Figure 2**). The first two principal components (PC1, PC2) cumulatively explained 93.8% (**Supplementary Figure 2B**) and 95.6% (**Supplementary Figure 2C**) of total phenotypic variation among rice accessions across different aging treatments at 14 and 28 days, respectively.

### 3.2 Effect of EPPO storage on seed longevity parameters

Germination data of the 20 rice seed lots following storage under EPPO as well as their pressure-corrected values (ΔEPPO) for different storage durations (up to 56 days) were fitted to estimate the seed longevity parameters of equation 1 (*K*_i_, σ^-1^) and *P*_50_, which showed clear differences among these lots (**Table 1** and **Supplementary Figure 3**). Under EPPO storage, after pressure effect correction (ΔEPPO), the initial seed viability (*K*_i_) among the 20 rice seed lots ranged between 0.82 NED (IRGC 117274) to 3.10 NED (IRGC 117283) with a mean of 1.68 NED (**Table 1**). The estimates of σ^-1^ ranged between 0.041 (IRGC 117267) to 0.097 (IRGC 117283) with a mean of 0.073 NED d^-1^. The *P*_50_ values also showed considerable variation among rice accessions when stored under EPPO: IRGC 117283 was the most tolerant with its longest *P*_50_ of 31.8 days, and IRGC 117274 was the most sensitive with a *P*_50_ of 12.0 days (**Table 1**). The observed considerable genetic variation for sensitivity to elevated oxygen levels in this panel of 20 diverse rice accessions indicates that EPPO storage can be a useful experimental aging test to estimate the relative shelf-life of rice seed lots.

**Table 1:** Seed longevity parameters derived from probit analysis of total germination data upon removal from storage for 20 rice accessions under dry-EPPO aging conditions in experiment 1.

Accession Nr.	Variety Name	EPPO			ΔEPPO		
		K*_i_* (s.e.)	σ ^–1^ (s.e.)	*P_50_* (days)	K*_i_* (s.e.)	σ^–1^ (s.e.)	*P_50_* (days)
IRGC 117264	AZUCENA	1.63 (0.12)	0.083 (0.005)	19.6	1.60 (0.12)	0.073 (0.005)	21.8
IRGC 117265	DOM SUFID	2.18 (0.18)	0.079 (0.007)	27.4	2.25 (0.18)	0.083 (0.006)	27.3
IRGC 117266	DULAR	1.42 (0.11)	0.069 (0.005)	20.7	1.66 (0.15)	0.072 (0.005)	23.1
IRGC 117267	FR 13 A	3.97 (0.50)	0.123 (0.015)	32.2	0.89 (0.11)	0.041 (0.004)	21.7
IRGC 117268	IR 64-21	1.58 (0.11)	0.068 (0.005)	23.5	1.78 (0.15)	0.072 (0.005)	24.5
IRGC 117269	LI JIANG XIN TUAN HEI GU	1.30 (0.11)	0.077 (0.005)	16.9	1.25 (0.12)	0.050 (0.004)	25.2
IRGC 117270	M202	1.45 (0.11)	0.098 (0.006)	14.7	1.12 (0.10)	0.061 (0.004)	18.4
IRGC 117271	MINGHUI 63	1.64 (0.12)	0.086 (0.006)	19.1	2.05 (0.15)	0.092 (0.006)	22.2
IRGC 117272	MOROBEREKAN	2.31 (0.19)	0.087 (0.007)	26.4	1.73 (0.15)	0.068 (0.005)	25.6
IRGC 117273	N 22	1.12 (0.09)	0.064 (0.004)	17.5	1.29 (0.10)	0.050 (0.003)	25.6
IRGC 117274	NIPPONBARE	0.70 (0.09)	0.080 (0.006)	8.8	0.82 (0.12)	0.068 (0.006)	12.0
IRGC 117275	POKKALI	1.98 (0.14)	0.079 (0.005)	25.1	2.36 (0.20)	0.095 (0.007)	24.9
IRGC 117276	SADU CHO	2.02 (0.14)	0.099 (0.006)	20.3	1.78 (0.13)	0.078 (0.005)	22.7
IRGC 117277	SANHUANGZHAN No 2	1.58 (0.09)	0.064 (0.003)	24.7	1.83 (0.15)	0.070 (0.005)	25.9
IRGC 117278	SWARNA	1.44 (0.10)	0.062 (0.004)	23.4	1.59 (0.12)	0.070 (0.004)	22.7
IRGC 117279	TAINUNG 67	1.50 (0.14)	0.101 (0.007)	14.9	1.23 (0.13)	0.071 (0.006)	17.3
IRGC 117280	ZHENSHAN 97 B	1.72 (0.13)	0.104 (0.007)	16.5	1.73 (0.16)	0.086 (0.007)	20.2
IRGC 117281	ASWINA	2.72 (0.19)	0.096 (0.006)	28.4	2.34 (0.19)	0.089 (0.007)	26.3
IRGC 117282	CYPRESS	1.70 (0.15)	0.119 (0.009)	14.3	1.26 (0.13)	0.071 (0.006)	17.9
IRGC 117283	RAYADA	3.24 (0.36)	0.102 (0.011)	31.8	3.10 (0.27)	0.097 (0.008)	31.8
	Maximum	3.97	0.123	32.2	3.10	0.097	31.8
	Minimum	0.70	0.062	8.8	0.82	0.041	12.0
	Mean	1.86	0.087	21.2	1.68	0.073	22.8

IRGC-International Rice Genebank Collection; EPPO-Elevated Partial Pressure of Oxygen; ΔEPPO-corrected EPPO values; K_*i*_- initial viability in NED; σ^–1^ -length of time for viability to fall by 1 NED; *P*_50_-length of time for viability to fall to 50%; s.e.- standard error.

### 3.3 Effect of EPPO on rice seed lipidome

**Figure 2:**
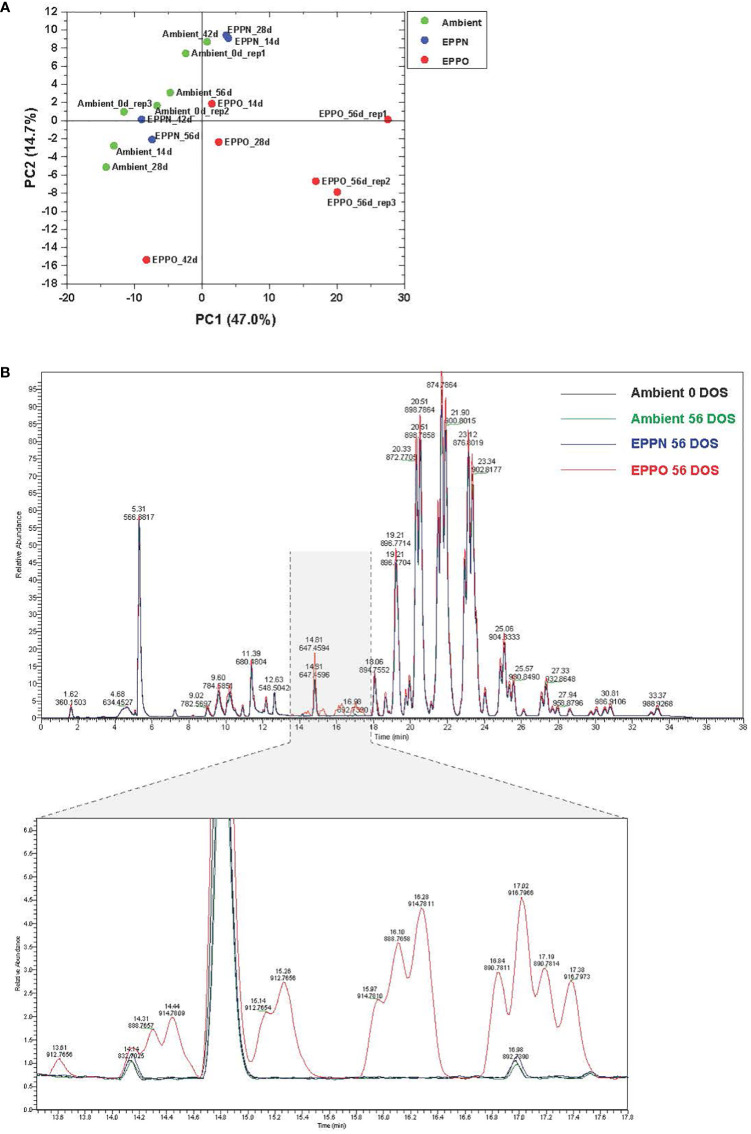
Lipid analysis of rice seeds stored under EPPO and its control conditions. **(A)** Principal component analysis based on the relative abundance of 300 lipid compounds. **(B)** Overlay of total ion chromatograms of several lipid species in rice seed samples from the ambient initial control (Ambient 0 DOS) or aged under different conditions for 56 days.

Since seed aging involves lipid oxidation processes, we studied the effects of EPPO treatment on the overall lipid composition in rice seeds from cv IR64 stored under EPPO and control conditions for different durations. The untargeted processing of the Liquid Chromatography-Mass Spectrometry (LC-MS) data resulted in the relative abundance values, corrected for the internal standard, of over 652 lipid-soluble compounds across all samples (**Supplementary Data 1**). Differences in their lipid profiles between seed samples stored under different storage conditions are depicted in **Figure 2** and **Supplementary Figure 4**. A PCA, based on the relative abundance of 300 reproducibly detected compounds (using the criterium that the compound should be present in at least three samples at a signal abundance >15,000, i.e., three times the set detection threshold for peak picking) clearly separated the rice seed samples stored under EPPO from those of ambient control and EPPN control treatments (**Figure 2A**). The first two principal components explaining approximately 62% of the total variation (PC1 = 47.0%, PC2 = 14.7%), indicate that the 56 DOS EPPO-stored samples have a distinctive lipid profile compared to the ambient or 56 DOS EPPN stored samples. This is in agreement with the contrasting germination performance after the treatments. Specifically, the abundance of a series of lipid compounds eluting between 13.40 and 17.60 minutes was highly characteristic for the stronger-aged EPPO stored seed samples (**Figure 2B**). The results of a Hierarchical Cluster Analysis (HCA), based on the same 376 lipid compounds, are represented as a dendrogram and heatmap (**Supplementary Figure 5**). The HCA confirmed that the profiles of the EPPO treated seeds have lipid profiles distinct from the two control treatments, with several lipid compounds specifically or much more substantially accumulating in the EPPO stored samples. There was no clear separate clustering of the two control treatments meaning that the EPPN treatment, which included a pressure induced ERH increase, did not result in apparent changes in the lipid profile compared to the storage at ambient pressure and ERH (**Supplementary Figure 5**).

### 3.4 Correlation of lipids from EPPO stored seeds with seed viability and storage duration

The metabolomics data were also explored to identify compounds correlating to seed viability and compounds accumulated during storage at each condition. Pearson’s correlations between the abundance of each lipid compound and the average percentage of seed germination of the respective samples were calculated (**Supplementary Data 1**). The topmost compounds showing the highest negative correlations (*r*< -0.98) and highest positive correlations (*r* > 0.85) were selected for further annotation based on the observed accurate mass and potentially present molecular fragments (**Table 2**). A negative correlation with seed viability, and thus positive with seed aging, was observed with compounds identified as oxidized lipids with one or more oxygen atoms incorporated (**Table 2**). The only exception was Triacylglyceride (TAG) 52:3+O (m/z [M+Na]^+^ = 890.7812, cluster ID 483 in **Supplementary Data 1**) eluting at retention time 17.5 min), which was highest at the initial time point (0 day) and decreased to non-detectable levels upon subsequent EPPO storage. Apparently, this mono-oxygenated unsaturated TAG was already present in initial seeds and was further oxidized and converted into one of the TAGs of other lipid compounds increasing by EPPO storage and negatively correlating with germination performance. Lipid compounds showing highest positive correlation with seed viability, with a decline during seed EPPO aging, were identified as the lipophilic antioxidant coenzyme Q9 and unsaturated lipids typically found in rice seeds (e.g., tri-linoleoyl-glycerol, cluster ID 440 in **Supplementary Data 1**).

**Table 2:** List of topmost lipids showing the highest correlation with seed viability (%) and storage time (days) in seeds of rice cv. IR64 stored under EPPO aging treatments.

Cluster ID	m/z	Retention time (min)	Adduct (H^+^ or NH4^+^)	Total formula	Elemental formula + # oxygen atoms added	Putative annotation	ID^a^	Percentage increase over initial control in EPPO aging treatment				r^Viability^	r^Storage time^
								14d	28d	42d	56d		
63	340.2842	4.7	H^+^	C_20_H_37_NO_3_	C_20_H_37_NO_2_ + 1 O	Anandamide + 1 O	LM	128.7	155.6	220.4	239.0	-1.00	0.98
24	348.2746	3.0	NH4^+^	C_18_H_34_O_5_	C_18_H_34_O_2_ + 3 O	Oleic acid + 3 O	LM	115.5	140.3	180.0	206.5	-0.99	0.99
57	279.2320	4.6	–	–	–	unknown	–	128.9	177.3	290.7	303.2	-0.99	0.97
346	804.6718	13.1	NH4^+^	C_49_H_86_O_7_	C_49_H_86_O_6_ + 1 O	TAG 46:4 + 1 O	7803	101.3	108.7	120.9	127.2	-0.98	0.97
353	906.7767	13.3	NH4^+^	C_55_H_100_O_8_	C_55_H_100_O_6_ + 2 O	TAG 52:3 + 2 O	8487	461.7	937.0	1232.6	1854.2	-0.98	0.99
571	738.6757	19.5	H^+^	C_49_H_84_O_3_	C_49_H_84_O_2_ + 1 O	CE 22:2 + 1 O	LM	189.6	260.3	340.2	483.8	-0.98	0.99
347	930.7766	13.1	NH4^+^	C_57_H_100_O_8_	C_57_H_100_O_6_ + 2 O	TAG 54:5 + 2 O	8506	640.6	1527.2	2028.5	3229.6	-0.97	0.99
577	947.8470	19.7	–	–	–	unknown	–	284.3	391.5	529.7	805.1	-0.97	0.99
447	888.7655	16.1	NH4^+^	C_55_H_98_O_7_	C_55_H_98_O_6_ + 1 O	TAG 52:4 + 1 O	7852	278.1	397.1	483.7	735.1	-0.96	0.99
499	892.7970	18.0	NH4^+^	C_55_H_102_O_7_	C_55_H_102_O_6_ + 1 O	TAG 52:2 + 1 O	7838	426.2	610.6	822.3	1341.2	-0.96	0.98
469	916.7968	17.0	NH4^+^	C_57_H_102_O_7_	C_57_H_102_O_6_ + 1 O	TAG 54:4 + 1 O	7855	306.1	426.5	514.1	791.3	-0.96	0.98
410	892.7982	15.0	NH4^+^	C_55_H_102_O_7_	C_55_H_102_O_6_ + 1 O	TAG 52:2 + 1 O	7838	261.5	383.1	451.8	697.4	-0.96	0.98
386	911.7534	14.3	–	–	–	unknown	–	303.9	470.9	549.1	859.1	-0.96	0.98
194	411.3625	10.1	–	–	–	unknown	–	108.4	108.9	113.4	116.7	-0.96	0.96
420	912.7656	15.3	NH4^+^	C_57_H_98_O_7_	C_57_H_98_O_6_ + 1 O	TAG 54:6 + 1 O	7867	282.5	409.8	482.8	748.6	-0.96	0.98
519	722.6447	18.3	H^+^	C_40_H_80_O_3_	C_40_H_80_O_2_ + 1 O	CE 21:3 + 1 O	LM	251.5	433.2	503.5	801.4	-0.96	0.98
453	914.7811	16.3	NH4^+^	C_57_H_100_O_7_	C_57_H_100_O_6_ + 1 O	TAG 54:5 + 1 O	7854	303.8	432.6	514.6	807.6	-0.96	0.98
388	919.7368	14.4	–	–	–	unknown	–	311.9	466.3	517.7	778.7	-0.96	0.98
129	800.5808	7.0	H^+^	C_51_H_74_O_6_	C_51_H_74_O_2_ + 4O	Prenol lipid, unknown	LM	379.6	483.2	592.9	917.1	-0.96	0.98
122	798.5648	6.6	H^+^	C_44_H_80_NO_7_P	C_44_H_80_NO_5_P + 2 O	PC 36:4 + 2 O	LM	443.6	660.1	728.2	995.1	-0.96	0.98
382	888.7656	14.3	NH4^+^	C_55_H_98_O_7_	C_55_H_98_O_6_ + 1 O	TAG 52:4 + 1 O	7852	279.8	422.8	490.6	789.2	-0.95	0.98
517	918.8124	18.2	NH4^+^	C_57_H_104_O_7_	C_57_H_104_O_6_ + 1 O	TAG 54:3 + 1 O	7857	302.0	420.6	500.6	800.6	-0.95	0.98
336	930.7764	12.8	NH4^+^	C_57_H_100_O_8_	C_57_H_100_O_6_ + 2 O	TAG 54:5 + 2 O	8506	335.1	611.3	709.2	1191.8	-0.95	0.98
342	910.7499	13.0	NH4^+^	C_57_H_96_O_7_	C_57_H_96_O_6_ + 1 O	TAG 54:7 + 1 O	7864	383.4	621.8	731.8	1223.0	-0.95	0.98
390	914.7811	14.5	NH4^+^	C_57_H_100_O_7_	C_57_H_100_O_6_ + 1 O	TAG 54:5 + 1 O	7854	346.8	543.1	624.5	1022.0	-0.95	0.98
433	862.7499	15.7	NH4^+^	C_53_H_96_O_7_	C_53_H_96_O_6_ + 1 O	TAG 50:3 + 1 O	7828	284.5	480.1	571.0	959.2	-0.95	0.98
423	916.7969	15.4	NH4^+^	C_57_H_102_O_7_	C_57_H_102_O_6_ + 1 O	TAG 54:4 + 2 O	7856	323.7	485.9	578.2	960.0	-0.95	0.98
366	912.7656	13.6	NH4^+^	C_57_H_98_O_7_	C_57_H_98_O_6_ + 1 O	TAG 54:6 + 1 O	7867	296.8	447.2	522.8	859.7	-0.95	0.98
481	916.7968	17.4	NH4^+^	C_57_H_102_O_7_	C_57_H_102_O_6_ + 1 O	TAG 54:4 + 1 O	7855	301.2	438.6	515.3	850.5	-0.95	0.98
425	891.7854	15.7	–	–	–	unknown	–	152.0	261.6	317.3	512.4	-0.95	0.97
489	943.8160	17.7	–	–	–	unknown	–	433.4	717.9	813.2	1374.9	-0.95	0.98
413	918.8130	15.2	NH4^+^	C_57_H_104_O_7_	C_57_H_104_O_6_ + 1 O	TAG 54:3 + 1 O	7857	577.9	843.3	949.5	1526.4	-0.95	0.98
415	887.7535	15.2	–	–	–	unknown	–	254.3	377.2	421.0	676.4	-0.95	0.98
476	890.7811	17.2	NH4^+^	C_55_H_100_O_7_	C_55_H_100_O_6_ + 1 O	TAG 52:3 + 1 O	7835	307.2	447.0	526.4	884.2	-0.95	0.97
524	892.7968	18.6	NH4^+^	C_55_H_102_O_7_	C_55_H_102_O_6_ + 1 O	TAG 52:2 + 1 O	7838	399.9	529.0	745.6	1307.1	-0.95	0.97
439	914.7811	16.0	NH4^+^	C_57_H_100_O_7_	C_57_H_100_O_6_ + 1 O	TAG 54:5 + 1 O	7854	300.7	420.9	487.8	806.8	-0.95	0.97
332	909.7162	12.5	–	–	–	unknown	–	230.7	488.1	626.4	1123.4	-0.95	0.97
472	921.7524	17.1	–	–	–	unknown	–	654.0	796.3	938.4	1367.3	-0.95	0.97
434	812.6558	15.7	H^+^	C_54_H_82_O_4_	–	Coenzyme Q9	LM	90.7	77.8	70.0	79.6	0.84	-0.83
157	955.7575	8.9	H^+^	C_61_H_108_O_6_	–	TAG 58:5	7256	97.4	85.6	80.2	86.7	0.84	-0.83
325	548.5040	12.6	H^+^	C_57_H_98_O_6_	–	TAG 54:6	7236	97.7	96.9	97.0	96.4	0.85	-0.88
143	190.1260	8.0	–	–	–	unknown	–	91.9	87.2	74.2	82.2	0.90	-0.86
467	892.7393	17.0	NH4^+^	C_57_H_94_O_6_	–	TAG 54:8	7231	95.8	91.4	77.1	84.5	0.91	-0.86

The annotation of lipid species belonging to triacylglycerides (TAGs) or diacylglycerides (DAGs) is provided as the total number of carbons in their fatty acid chains, number of unsaturated bonds and the number of oxygen atoms incorporated. The provided annotations are putative, based on the elemental formula deduced from the detected accurate mz values.

^a^ – LM refers to Lipid Maps (https://www.lipidmaps.org); numbers refer to IDs of oxidized lipids from Riewe et al., 2017.

### 3.5 Effect of EPPO on the headspace volatiles profile and its correlation with seed viability

**Figure 3:**
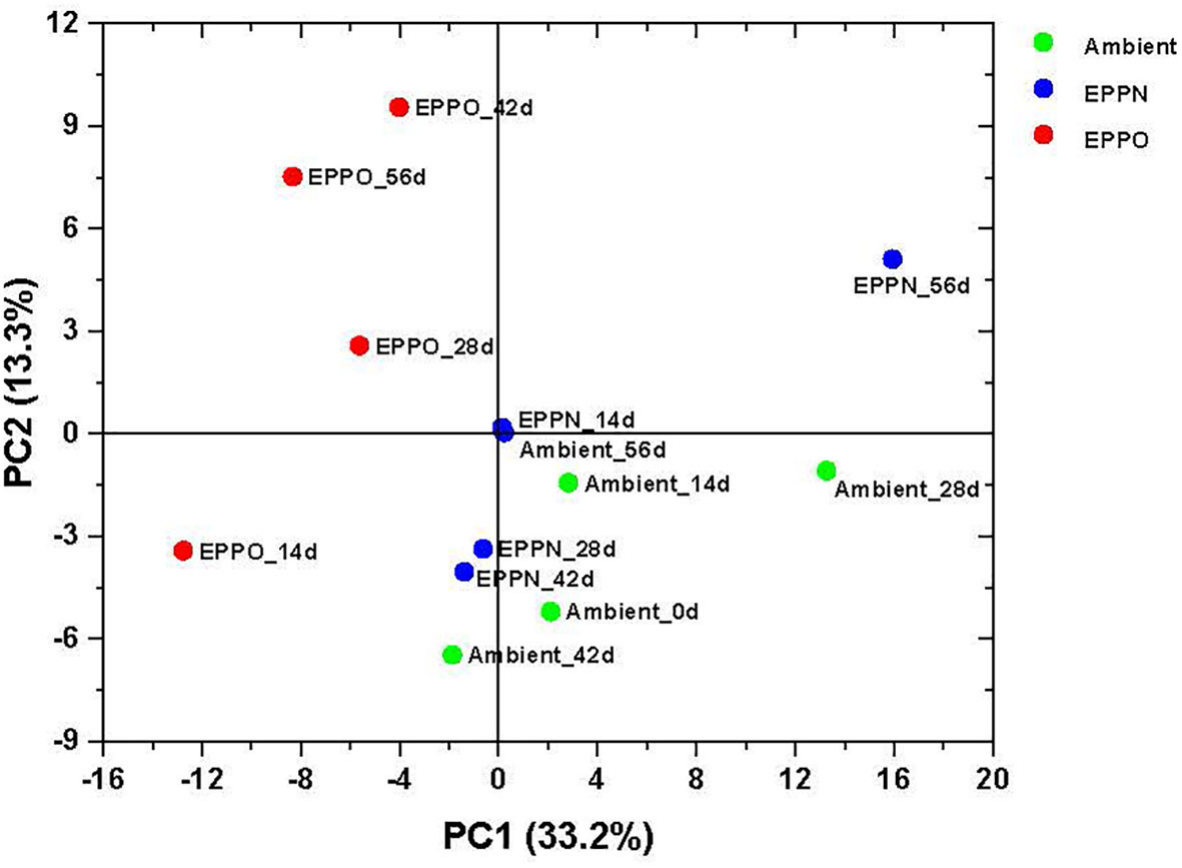
Headspace volatile analysis of rice seeds stored under EPPO and its control conditions. Principal component analysis based on the relative abundance of 183 volatile compounds.

The untargeted approach to profile volatile compounds using HS-SPME-GC-MS identified 224 compounds emitted from ground rice seed samples after storage under different aging treatments. This number was reduced to 183 reproducibly detectable compounds after applying strict filtering criteria (using the criteria that the metabolites are present in all the replications of the QC samples) (**Supplementary Data 2**). A PCA based on selected volatile compounds, where PC1 explains 33.2% of the total variation, clearly separates the samples aged under EPPO conditions from those aged under ambient and EPPN conditions (**Figure 3**). An HCA performed on the same data confirmed the deviating volatile profiles of the EPPO treated rice samples (**Supplementary Figure 6**). The heatmap showed the relative abundance of those volatile compounds accumulating over time, specifically in the EPPO stored samples.

Pearson’s correlation coefficients were calculated based on the relative abundance value of each volatile compound with percentage seed germination and storage duration (**Table 3**, **Supplementary Data 2**). Seeds stored under EPPO conditions were dominated by volatile compounds, which consistently and markedly increased as aging progressed, like 3,5-octadien-2-one, 2-methyl 2-propanol, hexanal, 2-heptanone, acetic acid, 2-butanone, heptanal, and 1-butanol.

**Table 3:** List of topmost headspace volatiles showing highest correlation with seed viability (%) and storage time (days) in seeds of rice cv. IR64 stored under EPPO aging treatments.

ClusterID	Retention time (min)	Annotation	Formula	Percentage increase over initial control in EPPO aging treatment				r*^Viability^*	r*^Storage time^*
				14d	28d	42d	56d		
161	16.7066	2,2,4,4-Tetramethyloctane	C_12_H_26_	241	344	500	538	-0.99	0.99
117	15.0172	5-Nonen-4-one, 6-methyl	C_10_H_18_O	151	266	384	476	-0.99	0.99
46	9.3585	2-Propanol, 2-methyl	C_4_H_10_O	240	776	1640	1986	-0.99	0.98
41	8.5192	Hexanal	C_6_H_12_O	196	276	380	376	-0.98	0.97
91	13.8115	2-Heptanone, 6-methyl	C_8_H_16_O	108	171	228	237	-0.98	0.97
185	17.7664	3,5-Octadien-2-one	C_8_H_12_O_2_	33861	116996	181865	300134	-0.97	0.98
101	14.3165	Benzaldehyde	C_7_H_6_O	184	240	410	371	-0.97	0.94
66	11.4534	Ethanone, 1-(1-cyclohexen-1-yl)	C_8_H_12_O	113	118	137	132	-0.97	0.94
4	3.2625	2-Butanone	C_4_H_8_O	214	187	367	340	-0.94	0.90
169	17.0942	3-Pentanol, 2,2-dimethyl	C_7_H_16_O	277	192	507	677	-0.94	0.92
188	17.8518	Acetophenone	C_8_H_8_O	93	125	137	151	-0.94	0.94
166	16.9911	2-Heptanone, 4,6-dimethyl	C_9_H_18_O	216	329	543	433	-0.93	0.90
17	4.6104	Benzene	C_6_H_6_	120	120	185	167	-0.93	0.88
30	6.8440	Disulfide, dimethyl	C_2_H_6_S_2_	235	280	378	686	-0.92	0.95
67	11.5565	2-Heptanone	C_7_H_14_O	96	124	228	200	-0.91	0.87
90	13.6550	2(5H)-Furanone, 5,5-dimethyl	C_6_H_8_O_2_	107	111	115	134	-0.91	0.94
42	8.8499	Cyclotrisiloxane, hexamethyl	C_6_H_18_O_3_Si_3_	99	100	107	110	-0.91	0.89
49	9.8102	2,2-Dimethyl-3(2H)-furanone	C_6_H_8_O_2_	107	185	255	214	-0.91	0.88
16	4.5429	1-Butanol	C_4_H_10_O	155	215	300	243	-0.91	0.88
73	11.9584	Cyclohexanone, 2-acetyl	C_8_H_12_O_2_	107	216	265	237	-0.91	0.89
205	18.7659	Î²-Myrcene	C_10_H_16_	381	29	2241	2043	-0.89	0.83
75	12.0225	Heptanal	C_7_H_14_O	162	239	237	242	-0.88	0.90
43	8.9140	Acetic acid, butyl ester	C_6_H_12_O_2_	145	636	565	705	-0.87	0.90
113	14.8713	5-Hepten-2-one, 6-methyl	C_8_H_14_O	86	102	143	137	-0.87	0.83
118	15.1345	Furan, 2-pentyl	C_9_H_14_O	106	107	164	143	-0.87	0.81
213	19.6764	2-Decanone	C_10_H_20_O	101	152	204	165	-0.86	0.83

### 3.6 Comparison of seed longevity under EPPO, CD and ambient long-term storage conditions

**Figure 4:**
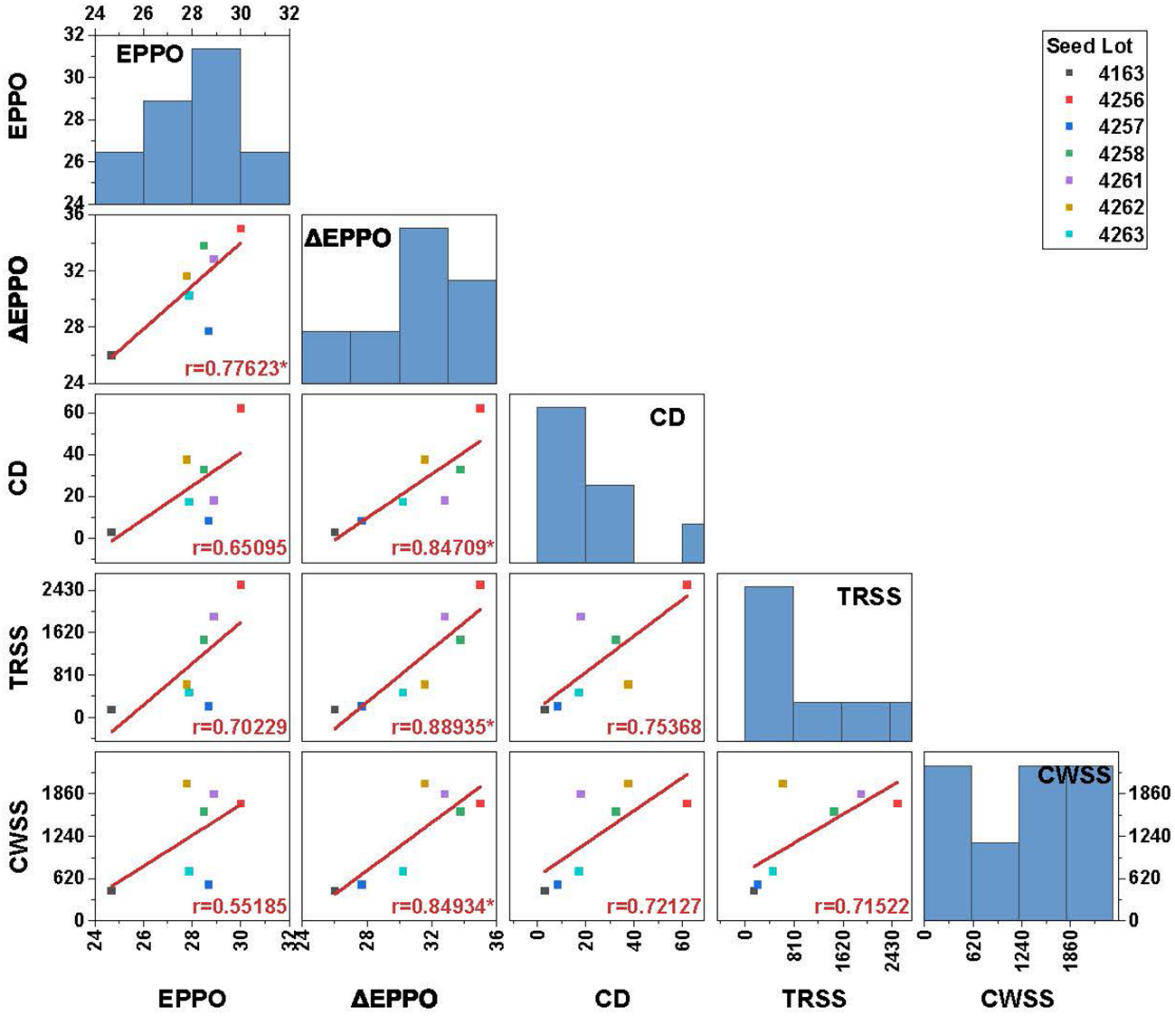
Pearson’s correlation coefficient matrix between *P*_50_ values for 7 different seed lots aged under long-term natural and experimental aging conditions. Bar chart in the plot along the diagonal indicates frequency distribution and the values in the X and Y-axis indicates storage duration in days. Significance codes: ‘*’ *p*<0.05. EPPO-Elevated partial pressure of oxygen; ΔEPPO-Corrected EPPO values; CD-controlled deterioration; TRSS-Traditional rice seed storage; CWSS-Conditioned warehouse seed storage; *P*_50_-time for viability to decrease to 50%.

To analyze to what extent dry EPPO aging mimics long-term natural aging, seeds of seven lots were also stored under conditions mimicking CWSS and TRSS. In addition, we compared these dry aging conditions with experimental aging at relatively high moisture conditions (CD test). Aging under both EPPO and CD treatment resulted in faster deterioration of the seeds, as shown in a decreased germination rate compared with aging under CWSS and TRSS conditions (**Supplementary Figure 7**). Under CWSS and TRSS conditions, three seed lot (4256, 4258, and 4261) showed a negligible change in germination performance, even after prolonged storage. The rate of seed viability loss (estimated as the duration to 50% viability loss, *P*_50_) under the different storage conditions was calculated for each seed lot using the seed viability equation (equation 1 in the *Materials and Methods* section; **Supplementary Table 4**). Correlation analysis based on *P*_50_ values showed that ΔEPPO is significantly (*p*<0.05) positively correlated with both TRSS (r=0.89), CWSS (r=0.85) and CD (r=0.85) conditions (**Figure 4**). The significant but relatively low correlation of 0.78 for *P*_50_ between EPPO and ΔEPPO indicates variation between seed lots in their sensitivity to high pressure (as deduced from the EPPN storage effects).

### 3.7 Comparison of seed metabolic profiles under EPPO, CD, and ambient long-term storage conditions

**Figure 5:**
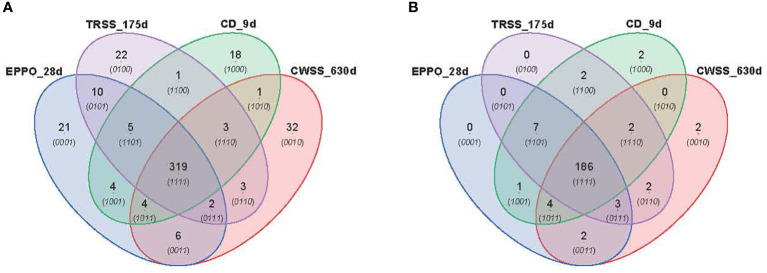
Comparison of overall lipid and volatile metabolites at storage time point when seed germination had decreased to around 50% (*P*_50_). **(A)** Venn diagram showing number of unique and overlapping lipid compounds. **(B)** Venn diagram for volatile compounds.

PCA was performed to compare the overall profiles of lipid-soluble compounds and headspace volatiles in rice seeds stored under the different aging conditions. The first two PCs together explained 47.4% and 54.5% of the total variation in the profiles for lipid compounds (**Supplementary Figure 8A**) and volatile compounds (**Supplementary Figure 8B**), respectively. Both PCA score plots did not show a clear separation of samples based on storage condition only, likely due to differential storage times imposing different levels of aging. A time-dependent effect is observed in these PCAs except for the samples aged under CD conditions. A more detailed comparison between the aging conditions was made for those samples closest to the storage time resulting in 50% viability (*P*_50_), i.e., 28d for EPPO, 9d for CD, 175d for TRSS, and 630d for CWSS. In total, 319 lipid compounds (**Figure 5A**) and 186 headspace volatile compounds (**Figure 5B**) were commonly detected across all four storage conditions at the *P_50_* time points. This considerable overlap may relate to the observed lack of storage-related clustering of samples in the unsupervised PCA (**Supplementary Figure 8**). At their respective *P*_50_ effective time points, EPPO-stored seeds contained 21 unique lipid compounds, the CD contained 22, TRSS 18, and CWSS 32 lipid compounds (**Figure 5A**). Similarly, only two volatile compounds were unique to the CD storage and two others for CWSS (**Figure 5B**).

**Figure 6:**
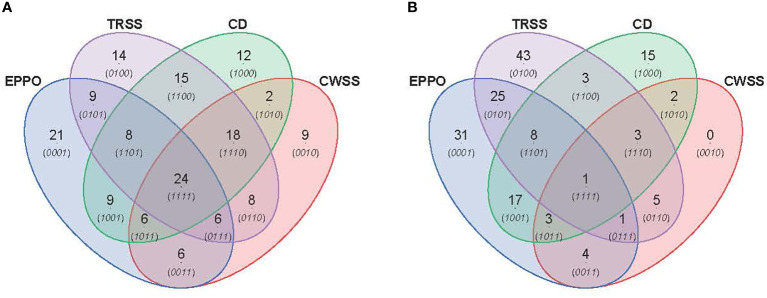
Comparison of lipid **(A)** or volatile **(B)** compounds detected in aged rice seeds stored under different aging treatments. Lipid or volatile compounds are depicted, with a negative correlation (*r*>0.50) for germination percentage after storage.

The metabolomics data were also explored to identify compounds correlating to the loss in seed viability and accumulation during storage at each aging condition (**Figure 6**). A total of 89, 102, 94, and 79 lipid compounds in seeds stored under EPPO, TRSS, CD, or CWSS conditions, respectively, showed a high negative correlation (*r* > -0.50) with total percentage germination (**Supplementary Data 1**), while for the volatile compounds these values were respectively 90, 89, 52, and 17 (**Supplementary Data 2**). These correlating compounds were visualized by a Venn diagram to find unique and overlapping metabolites across all four storage conditions (**Figure 6**). Interestingly, 24 lipid compounds and only one volatile compound were common to all four storage conditions, while 21, 14, 12, and 9 lipid compounds (**Figure 6A**) and 31, 43, 15, and 0 volatile compounds (**Figure 6B**) were found unique to EPPO, TRSS, CD, and CWSS conditions, respectively. Based on these correlating lipid compounds, EPPO storage had the highest overlap with both TRSS and CD (9 compounds each), followed by CWSS (6 compounds) (**Figure 6A**). Similarly, for the correlating volatile compounds, EPPO storage had the highest overlap with compounds found under TRSS (25), followed by CD storage (17) and CWSS (4) (**Figure 6B**).

The topmost lipid and volatile compounds showing the highest negative correlation with the percentage of seed germination were selected for further manual annotation based on their mass spectrometry data (**Supplementary Table 5**, **6**). Most lipid compounds in seed samples stored under EPPO (**Supplementary Table 5A**) and CD conditions (**Supplementary Table 5D**) were identified as oxidized lipids with one or more oxygen atoms incorporated. Interestingly, lipids annotated in seeds stored under the two ambient long-term storage (CWSS & TRSS) showed a different lipid landscape compared to the two-fast aging assays (**Supplementary Table 5B, C**). Similarly, 3,5-octadien-2-one was among the major volatile compounds in seeds aged under EPPO conditions, which consistently increased many folds as aging progressed. 1-Butanol and acetic acid were found in seeds aged under EPPO, CWSS, and TRSS conditions but not in the CD samples (**Supplementary Tables 6A–C**). Major volatiles specific to CD storage included naphthalene and various benzene derivatives (**Supplementary Table 6D**).

## 4 Discussion

Before a seed lot completely loses the ability to germinate, damage accumulates because of aging. This damage reduces seed vigor, visible by slower germination and delayed seedling emergence, which can affect agricultural production (Finch-Savage and Bassel, 2016). Drying, to reduce the seed moisture content, and subsequent storage at low temperatures can substantially extend shelf-life, compared to storage at ambient conditions. However, monitoring shelf-life under dry storage conditions requires experiments lasting several years up to decades (Walters et al., 2010). The low correlation between aging under long-term natural, dry storage conditions and frequently used experimental moist aging conditions, applied to speed up the aging processes (Agacka-Mołdoch et al., 2016; Colville and Pritchard, 2019) calls for an improved experimental method that better mimics seed aging under long-term dry storage. Previously, Groot et al. (2012) demonstrated that for several non-related plant species, seed deterioration under dry conditions could be accelerated by storing seeds at normal temperatures but at higher oxygen concentrations, called “EPPO” storage. Our purpose here was to validate this EPPO storage method as a dry experimental aging tool for rice seeds and subsequently identify the underlying chemical changes induced by EPPO storage (35°C and RH of 50%) as compared to moist accelerated aging (CD) tests and to natural long-term dry storage conditions in practice in rice production areas. The obtained results showed that (1) seeds of rice deteriorate faster under EPPO compared to ambient storage conditions (2) different rice accessions exhibit variable germination responses to dry EPPO storage (**Figure 1** and **Table 1**); (3) EPPO storage significantly alters seed lipid and volatile profiles, and especially decreases the levels of unsaturated lipids, while increasing the levels of various oxidized lipid species (**Figure 2** and **Table 2**) and specific volatiles (**Figure 3** and **Table 3**), which are negatively correlating with the proportion of germinating seeds; (4) based on *P*_50_ values, a few weeks of EPPO storage at 50% ERH essentially mimics aging for more extended periods under ambient pressure at 40 or 60% RH and to a lesser extent CD aging at 75% RH (**Figure 4** and **Supplementary Table 4**); and (5) based on selected lipid and headspace volatiles showing high negative correlation with the proportion of viable seeds, aging under EPPO storage at 50% RH is more closely related to aging under long-term ambient storage at 60% RH (TRSS) or CD aging than to CWSS conditions with 40% RH (**Figures 5**, **6**). These salient findings are discussed below.

### 4.1 Seed aging under ambient and pressure control conditions

The principle of experimental aging by EPPO is the acceleration of oxidation-induced damage through elevation of oxygen availability rather than aging by increasing moisture and temperature. A disadvantage of EPPO is that the increased pressure will theoretically increase the water activity (a_w_) in seeds and that the build-up and decline in pressure, before and after the actual EPPO storage, can potentially induce physical damage to the seed. To correct for this physical pressure effect, the EPPN treatment was used as a control next to storage at ambient air pressure. With the EPPN treatment, the seed water activity will be similarly increased as with the EPPO treatment. Therefore, we expected that the EPPN treated seeds at 50% RH would age somewhat faster compared to ambient pressure stored seeds storage at 43% RH (**Figure 1**). We observed that seeds from some accessions aged somewhat faster under the EPPN treatment compared to the ambient control, specifically regarding their slower germination (increase in t_50_), while for other accessions there was no significant difference (**Supplementary Figure 3**).

In contrast, there was no significant difference between ambient and EPPN controls for average total seed germination and seedling quality parameters (**Figure 1**). Adverse effects of EPPN storage on seeds, resulting in a higher proportion of abnormal seedlings, have been reported for barley, with significant genetic variation for this sensitivity (Nagel et al., 2016). Since the observed negative effects of EPPN storage for some accessions depended on storage duration, it seems to be a direct or indirect physiological or biochemical effect during storage, rather than physical damage upon pressure build-up or pressure release at the start and end of EPPN storage, respectively. The theoretical slightly higher water activity induced by the higher pressure could be a contributing factor, but an effect of a high nitrogen pressure itself cannot be ruled out. An anesthetic effect by the increased partial pressure of nitrogen and some other gasses is well known with SCUBA diving, and their relative effect seems to be related to the gas lipid solubility (Bennett, 2004). The air used in the EPPO storage contains 78% nitrogen, resulting in a partial nitrogen pressure of 15.6 MPa, which approaches the 20 MPa in the EPPN treatment. Therefore, changes in germination parameters due to the increase in pressure were corrected in the EPPO treatment and represented as “ΔEPPO”, which is used to discuss the results observed in different experiments.

### 4.2 Rice seed aging under EPPO storage

Our experiments with rice seeds under dry EPPO showed increased aging during storage, resulting in the typical symptoms of seed viability loss: slower germination and a decline in normal seedlings (**Figure 1** and **Supplementary Table 3**). It also confirms the negative effect of oxygen on rice seed shelf-life (Roberts, 1961), as was observed with other species (Justice and Bass, 1978; Schwember and Bradford, 2011; Groot et al., 2015). Our results are consistent with other studies using EPPO as an experimental dry aging method (Groot et al., 2012; Nagel et al., 2016; Buijs et al., 2020; Hourston et al., 2020; Renard et al., 2020) or older experiments on seed storage under increased oxygen pressure (Ehrenberg et al., 1957; Moutschen-Dahmen et al., 1959; Berg et al., 1965). Accelerated after-ripening under EPPO conditions, as previously reported for *Arabidopsis* (Buijs et al., 2018; Buijs et al., 2020), was also observed for some seed lots in our experiments (**Supplementary Figure 3**). This dormancy loss was faster under EPPO storage compared to storage under ambient pressure or EPPN conditions, indicating a role of oxygen-dependent reactions in the after-ripening process.

Another important observation from our study is that the tested seed lots, representing different rice accessions, showed considerable variation in viability loss under EPPO storage (**Figure 1A**), indicated by the estimated *P*_50_ values (**Table 1**). Although seed production and handling can influence subsequent shelf-life, we expect that the variation among the analyzed seed lots mainly reflects an interaction between the storage environment and genetics, as these seeds were produced at the same time and processed, handled, and stored similarly prior to our experiments. Large genetic plasticity for seed germination traits under EPPO aging was also found in barley (Nagel et al., 2016) and *Arabidopsis* (Buijs et al., 2020; Renard et al., 2020), where specific QTLs were identified. However, the analysis of genetic variation was not the aim of the experiments reported here.

Lipids are key molecules for energy storage, membrane functioning, and signaling. Lipid oxidation reactions induced by free radicals or ROS during storage are reported to be a primary cause of aging-related damage in seeds (Harman and Mattick, 1976; Johnson and Decker, 2015). In rice seeds, TAGs are the principal lipids (Morrison, 1995). These storage lipids are present in the form of lipid droplets or spherosomes in the aleurone layer, sub-aleurone layer, and embryo of rice seed (Juliano, 1983; Chapman and Ohlrogge, 2012). In the endosperm, these lipids are bound to protein bodies and starch granules. The fatty acid composition of rice seeds constitutes around 75-80% of unsaturated fatty acids, namely oleic acid (C18:1), linoleic acid (18:2), and linolenic acid (C18:3) (Tong et al., 2019). Considerable changes in lipid profiles, i.e. a decrease in the levels of both unsaturated fatty acids and phospholipids and an accumulation of lipid oxidation products like malondialdehyde (MDA), lipid peroxides, and oxidized TAGs, have been reported in response to rice seed aging (Gayen et al., 2014; Huang et al., 2014) and botanically related wheat seeds (Riewe et al., 2017). To obtain a comprehensive overview of changes in the seed lipidome, we performed an untargeted high-mass resolution LC-MS based lipidome comparison of dehulled rice seed samples stored for different durations. However, our goal was to identify only the main significantly altered lipid species rather than annotate as many detected compounds as possible. Different species from various lipid classes were represented in our rice seed samples, characterized by specific retention times and accurate masses of molecular ions ([M+H]+ or [M+NH3]+) (**Supplementary Data 1**). These included TAGs, phospholipids, glycolipids, and free fatty acids and their oxidized forms. Evaluation of these lipidomics data using PCA and HCA could separate experimentally aged EPPO samples from less aged control samples (**Figure 2A**, **Supplementary Figure 5**). The EPPO-aged seed samples had a relatively high abundance of oxidized lipid compounds (**Figure 2B** and **Table 2**), some of which were previously reported to accumulate in long-term dry stored wheat seeds (Riewe et al., 2017). By analyzing seed samples taken at increasing time under EPPO storage, we showed that these oxidized lipids negatively correlate with the proportion of germinating seeds and positively correlate with storage duration (**Table 2**). Due to the presence of unsaturated bonds in their molecular structures, polyunsaturated lipids are more susceptible to oxidative damage than saturated species. The oxidized lipids in our dry-aged EPPO seed samples are most likely the result of a non-enzymatic oxidation reaction (Oenel et al., 2017). However, enzymatic conversion by the action of lipid-soluble enzymes cannot be ruled out. For example, lipases that catalyze the hydrolysis of TAGs/diacylglycerides (DAGs), releasing fatty acids and glycerol, are stable and can still be active in dry wheat seeds even at low moisture levels (Barros et al., 2010; Wiebach et al., 2020). Because of the extremely low molecular mobility in dry seeds, such lipases should be close to their substrates. Although rice lipids will be exposed to lipases during seed grinding (Lam and Proctor, 2003), in our study, such potential lipase activity was prevented by grinding the seeds in liquid nitrogen and directly mixing the frozen powders with the organic extraction solvent. Interestingly, a single oxidized lipid species, i.e., TAG 52:3+O, showing the highest correlation with rice seed aging was also present in the initial seed material and thus might at least partly have been generated during seed processing and storage before the start of our experiments. Upon subsequent storage, such partially oxidized lipid may undergo further oxidation, e.g. into TAG 52:3+2O (m/z [M+Na]^+^= 906.7767 eluting at a retention time of 13.3 min).

The oxidative degradation of lipids was accompanied by the formation of lipid oxidation-related volatiles (**Table 3**, **Supplementary Data 2**), as has also been reported for seed aging in other crops (Mira et al., 2010). Also, with the volatile compounds as variables, the PCA discriminated seed samples stored under EPPO conditions from those stored under ambient or EPPN conditions (**Figure 3A**). A considerable number of lipid-derived volatile compounds accumulated more than 200-fold during EPPO storage and showed a strong negative correlation to loss in seed viability after the EPPO storage experiment (**Table 3**). These correlating volatiles included compounds putatively identified as hexanal, heptanal, 2-heptenal, 2-heptanone, which are typical oxidation products of unsaturated fatty acids such as oleic acid, linoleic acid and linolenic acid (Wilkens and Lin, 1970; Frankel, 1983; Lam and Proctor, 2003). Similarly, several esters (for example, acetic acid-butyl ester that accumulated during EPPO storage) are among the oxidation-related degradation products from unsaturated fatty acids (Goicoechea and Guillén, 2014).

This untargeted lipid and volatile analyses proved that dry rice seeds stored under EPPO conditions (high oxygen levels) are sensitive to lipid oxidation reactions. These lipid oxidation reactions will also involve membrane lipids and are most likely the main reasons for viability loss. Additional oxidative reactions, not studied in the present experiments resulting in oxidative damage to DNA, RNA or proteins, can also be expected under EPPO conditions (Ohlrogge and Kernan, 1982; Farmer and Mueller, 2013). Further research is required to verify the oxidative stress-related changes in proteins or nucleic acids related to seed aging under EPPO storage conditions.

### 4.3 Comparison of aging under EPPO with other storage conditions

In general, longevity increases with a decrease in seed moisture content and temperature in a quantifiable and predictable way (Roberts, 1973; Ellis and Roberts, 1980). Seed moisture content, storage temperature, and chemical composition determine the physical state of the aqueous and lipid domain of the cytoplasm, and thereby the biophysical and biochemical processes related to seed deterioration (Vertucci and Roos, 1990; Ballesteros et al., 2020). Under dry conditions, the cytoplasm is in a glassy state with extreme low molecular mobility, while under humid conditions, the cytoplasm becomes liquid, increasing molecular mobility and allowing enzyme activity (Gerna et al., 2022). It is interesting to know if and how aging under EPPO storage may mimic aging under long-term natural conditions. However, ‘natural aging’ is not unambiguously defined, as it ranges between storage conditions that exist in highly controlled environments, like gene banks and commercial storage facilities for high value vegetable seeds, to rather uncontrolled environments such as in warehouses for relatively low value cereal seeds and open storage by farmers. In many studies, natural aging refers to laboratory bench storage for which storage conditions are less-well defined or not described at all. The RH in a laboratory can vary considerably over time. To perform a direct comparison, we stored rice seeds of seven different lots under four defined conditions. The conditions were EPPO storage (200 times higher oxygen levels, 35°C and theoretically at 50% RH), ambient pressure (air) conditions mimicking medium-term seed storage under dry conditions in controlled warehouses (CWSS, seed equilibrated to 40% RH and 28°C), short-term traditional storage practice (TRSS, seeds equilibrated to 60% RH and 28°C) and CD storage (seeds equilibrated to 75% RH and 35°C). The duration of storage in which the seed lot viability declines to 50% (*P*_50_) is considered a suitable measure of seed longevity (Hay et al., 2022a). Based on the *P*_50_ values (**Supplementary Table 4**), it was observed that seeds from all lots lost their viability in shorter times under dry-EPPO storage or wet-CD storage compared to storage under the two ambient storage conditions (CWSS and TRSS). Results of correlation analysis for the *P*_50_ values for the seven seed lots indicated that high-pressure oxygen storage (ΔEPPO) showed significant (*p<*0.05) positive correlations with all the other storage conditions, while such significant correlations were not found between CD aging and either TRSS or CWSS nor between TRSS and CWSS (**Figure 4**). It is worth noting that although the seed lots showed less variation for the slope of aging under EPPO storage, significant differences between seed lots were still detected (**Supplementary Table 4** and **Supplementary Figure 7**). Groot et al. (2012) showed that storage of dry lettuce seeds under EPPO conditions (at 20 MPa P_O2_ and 40% RH) mimics dry aging symptoms as seen upon long-term storage in seed company warehouses (at ambient pressure and 30% RH) and that aging under EPPO conditions is physiologically different from aging under moist warm moist conditions at ambient pressure. They showed that in cabbage seeds, the level of tocopherol (vitamin E, a lipid-soluble antioxidant) linearly decreased with viability loss under EPPO storage but was more or less constant under CD storage at 85% ERH. A recent study using a diverse panel of *Arabidopsis* ecotypes showed that laboratory bench seed aging (40-60% RH, 20-25°C and 18 months) better correlates with a CD test (75%RH, 38°C & 14days; r=0.68) and EPPO (40% RH, 20°C & 5 months; r=0.47) compared to an accelerated aging test (100% RH, 39°C & 48 h; r=0.11) (Renard et al., 2020). In a QTL analysis with *Arabidopsis*, Buijs et al. (2020) compared EPPO storage at 20 MPa air after equilibration of the seeds at 35 or 55% RH (during the test likely 40 or 64% RH, when corrected for the theoretical pressure effect on water activity according to Okos et al. (2019)), with both CD-aging at 85% RH and lab bench-aging (undefined), and showed that the four storage conditions resulted partly in overlapping, partly in distinct QTLs. Also within these two EPPO treatments, both common and distinct QTLs were identified. Those results indicate that the response of the seeds is also influenced by the RH during the EPPO storage, as it is in other storage conditions.

In our EPPO treatment, the rice seeds were pre-equilibrated at 20°C and 40% RH, at which moisture level, we assume the seeds are still in a glassy state and metabolically inactive (Sun, 1997; Ballesteros et al., 2020; Hay et al., 2022b). In the EPPO system, aging occurs in closed chambers filled with high-pressure dry air and with silica gel pre-equilibrated at the same RH as the seeds to provide a buffer for any change in the seed moisture level due to filling with dry air (Bartolotta et al., 1991). According to Okos et al. (2019) the increased pressure (at 20 MPa) will, combined with the temperature effect on the water activity, give a theoretical increase in the water activity from 0.43 to 0.50, in accordance with 50% ERH. Unfortunately, our data loggers were not able to record the actual RH in the tank under pressure. Therefore, it still needs to be elucidated whether there is an actual and substantial increase in water activity during EPPO treatment. However, even when this is true, we presume that the cytoplasm in the embryo of rice seed at 50% RH and 35°C is not in the liquid state. This is supported by the observation of an apparent effect of increased oxygen levels on seed aging, which is not expected for seeds with a liquid cytoplasm (Gerna et al., 2022).

### 4.4 Changes in metabolite profiles in seeds aged under EPPO and other storage conditions

In this study, we also analyzed the biochemical changes in the seeds, as deduced from untargeted lipidomics and headspace volatile analyses. We observed that seed samples at the start of storage (0 DOS) for each of the four storage conditions did not cluster together. This observation may indicate already some metabolic changes induced during the preparation of the samples for the different storage treatments, which is mainly a temporary change in seed water activity for a few days. Upon subsequent storage-induced and aging to a more or less similar germination percentage (*P*_50_), we observed a large overlap in the presence of lipid species (319 out of the 652 compounds in total) and headspace volatiles (186 out of the 224) between all four storage conditions (**Figure 5**), indicating that many of the metabolic changes are common for all four aging conditions. These metabolites are mainly concerned with oxidized lipids. A more detailed analysis of compounds correlating (r< -0.50) in their relative abundance with loss in seed viability indicated that EPPO samples showed the highest overlap with samples aged under TRSS, followed by CWSS and CD storage (**Figure 6**). However, there was hardly an overlap between the most humid CD storage and the least humid CWSS storage for either lipids or volatiles, indicating apparent differences between both storage regimes in inducing biochemical changes in the seed (**Figure 6**). Chemical identification of the topmost correlating lipids indicated an increase in oxidized TAGs in both EPPO and CD samples compared to seeds stored under the two ambient storage conditions (**Supplementary Table 5**). The possible reason could be the warmer conditions used in the experimental aging conditions (35°C) compared to the temperature at TRSS and CWSS (28°C). The accumulation of oxidized lipids coincides with the reduction of unsaturated TAGs, most prominently in the EPPO-aged samples. It is important to note here that in TRSS and CWSS samples, the accumulation of oxidized lipids involved a lower number of different TAG species (**Supplementary Table 5B, C**). Increased levels of oxidized lipids have also been observed with wheat seeds stored at around 6% RH and 20°C temperature conditions compared to seeds stored at 13% RH at 0°C, followed by -18°C conditions (Riewe et al., 2017; Wiebach et al., 2020). CD-aged samples contained more oxidized DAGs in addition to TAGs, presumably due to the hydrolytic cleavage of (oxy)PLs (Riewe et al., 2017).

Relative levels of 1-butanol (a volatile compound identified in oxidized soybean oil (Frankel, 1983)) were increasing while germination rates decreased in both EPPO, CWSS, and TRSS samples, but not in CD samples (**Supplementary Table 6**). Similarly, acetic acid, a product of the Maillard reaction (Mancilla-Margalli and López, 2002), also consistently increased with loss in seed viability in EPPO, CWSS, and TRSS samples, but it showed an opposite trend in CD samples (r=0.85). Hexanal, a secondary product of linoleic acid oxidation (Lam and Proctor, 2003; Colville et al., 2012), was only found to increase as aging progressed in EPPO samples. The naphthalene and benzene derivatives observed among the volatiles from seeds stored under CD conditions were unexpected. They might be considered as contamination from the CD test performed in laminated foil pouches. In our study, both the lipidomics and headspace volatile analyses showed common and distinct biochemical changes in seeds aged under ambient (CWSS and TRSS) conditions (**Figure 6**). Taking the metabolite analyses together, under all four storage conditions tested, many of the accumulating metabolites are common and mainly lipid oxidation products, showing oxidation as a main deteriorating effect during seed aging. The correlation analysis showed that regarding the lipids or volatile metabolite pattern, EPPO storage at 50% ERH resembles better to TRSS at 60% compared to CWSS at 40% ERH and CD at 75% ERH, indicating a clear effect of ERH in a more detailed look on the different lipid oxidation products that are accumulating. The latter will be a result of production and downstream conversion.

## 5 Concluding remarks

The use of practical aging methods to assess and estimate the shelf-life of seed lots and to understand aging and tolerance processes are important issues in seed technology research. In this study, we optimized a fast dry aging method for rice seeds based on elevated oxygen conditions (EPPO) rather than elevated moisture and temperatures. Our results revealed considerable variation among diverse rice accessions for seed viability in response to EPPO storage. Our untargeted metabolomics approaches indicated clear changes in seed lipidome and headspace volatile signatures upon aging under EPPO conditions, with an increased relative abundance of several volatile and non-volatile lipid oxidation products highly correlating with the decline in seed viability. This increase in lipid oxidation products by EPPO is in line with the described effect of oxygen and free radicals on seed aging. The results suggest a crucial role for antioxidant systems in seeds as a protection mechanism to maintain vigor and viability during dry storage. Based on correlation analysis of germination data (*P*_50_ values) from our comparative storage study involving seven different rice seed lots, we conclude that aging under EPPO conditions is more closely related to dry aging under TRSS and CWSS than under moist CD conditions. However, our current experimental setup and analytical approaches did not yet clarify on how EPPO-dry aging is related to dry-aging under ambient storage. Further research specifically focused on this question and using more replicate samples, allowing more sophisticated statistics, is needed. Further research to analyze antioxidant mechanisms during rice seed aging under various storage conditions should provide more clues and may also aid in developing strategies to reduce vigor decline during rice seed storage. Since elevated oxygen levels induce fast aging reactions in dry rice seeds, the EPPO method has two main implications. Firstly, EPPO storage can be used as a fast experimental aging method to study the genetics of seed longevity under dry conditions. Secondly, seed storage at anoxia conditions may have large practical utility in order to extend the shelf-life and maintain the quality of high-volume cereal seeds in tropical and sub-tropical countries, where low moisture conditions for storage are hard to achieve, to ensure long-term food security and seed conservation.

## Data availability statement

The original contributions presented in the study are included in the article/**Supplementary Material**, further inquiries can be directed to the corresponding author.

## Author contributions

MP, GA, FH, and SG conceived and designed the research. MP, JK, SS, RV, and RM performed the experiments. MP, JK, RV, and SG analyzed data. MP, GA, FH, RV, RM, and SG wrote the manuscript. DK reviewed the manuscript. All authors contributed to the article and approved the submitted version.

## Funding

This research was funded through the Netaji-Subhas ICAR International Fellowship from Education Division, Indian Council of Agricultural Research, New Delhi and additional financial support from Wageningen Seed Centre, the Netherlands

## Conflict of interest

The authors declare that the research was conducted in the absence of any commercial or financial relationships that could be construed as a potential conflict of interest.

## Publisher’s note

All claims expressed in this article are solely those of the authors and do not necessarily represent those of their affiliated organizations, or those of the publisher, the editors and the reviewers. Any product that may be evaluated in this article, or claim that may be made by its manufacturer, is not guaranteed or endorsed by the publisher.

## Abbreviations

AA, Accelerated aging test; ANOVA, Analysis of variance; a_w_, Water activity; CD, Controlled deterioration test; CWSS, Conditioned warehouse seed storage; DOS, Days of storage; DAI, Days after imbibition; DAG, Diacylglyceride; DG, Diradylglycerolipids; EPPO, Elevated partial pressure of oxygen; EPPN, Elevated partial pressure of nitrogen; ERH, equilibrium relative humidity; FA, Fatty acids/esters; G_MAX_, Maximum germination; h, hours; HCA, Hierarchical Cluster Analysis; IRGC, International Rice Genebank Collection; IRIS, International Rice Information System; IRRI, International Rice Research Institute; L, Length; mm, millimeter; MG, Monoradylglycerolipids; MPa, Megapascal; PCA, Principal Component Analysis; P_O2_, Partial pressure of oxygen; *P*_50_, Length of time for viability to fall to 50%; ROS, Reactive oxygen species; RT, Room temperature; TAG, triacylglyceride; TG, Triradylglycerolipids; TNS, Total normal seedlings; TRSS, Traditional rice seed storage; t_50_, Time to 50% of the maximum germination; UFA, Unsaturated fatty acids; W, Width.
