# Supplementary figures and images for: Experimental rice seed aging under elevated oxygen pressure: Methodology and mechanism
Source: Front Plant Sci. 2022 Dec 1;13:1050411. doi: 10.3389/fpls.2022.1050411 (PMC9751813; doi:10.3389/fpls.2022.1050411)

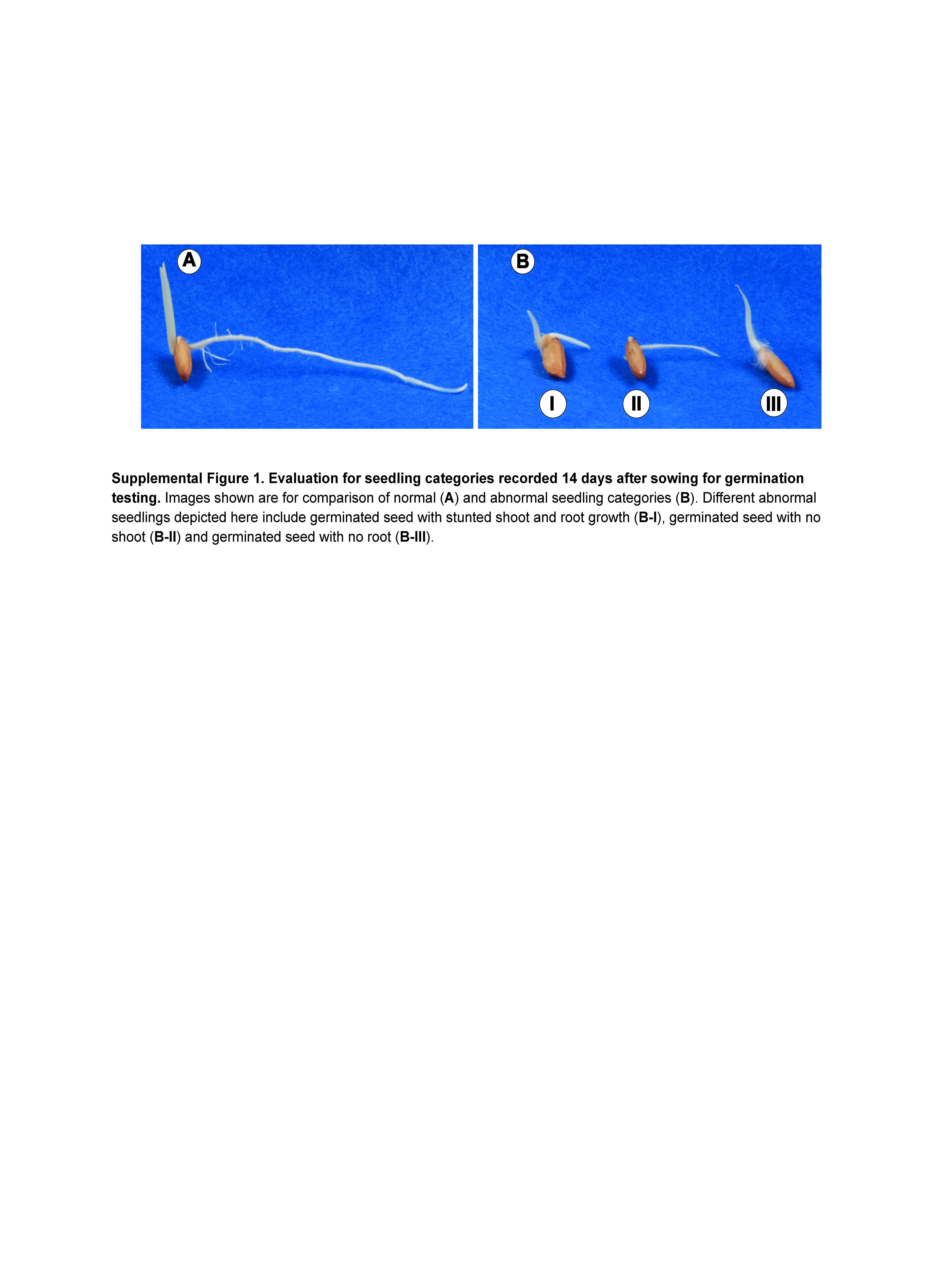

Supplement: Supplementary file 3 [file Image_1.jpeg]

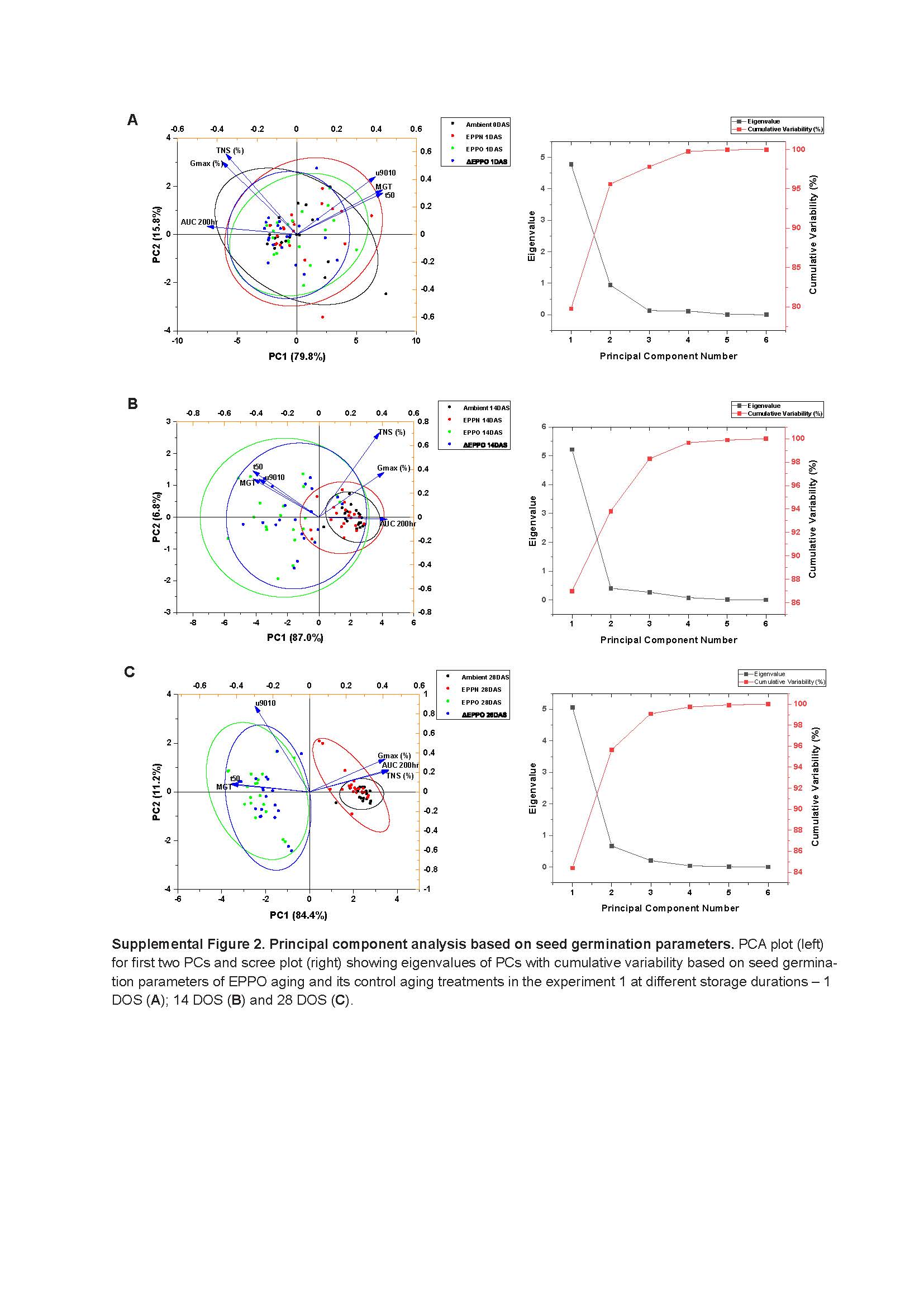

Supplement: Supplementary file 4 [file Image_2.jpeg]

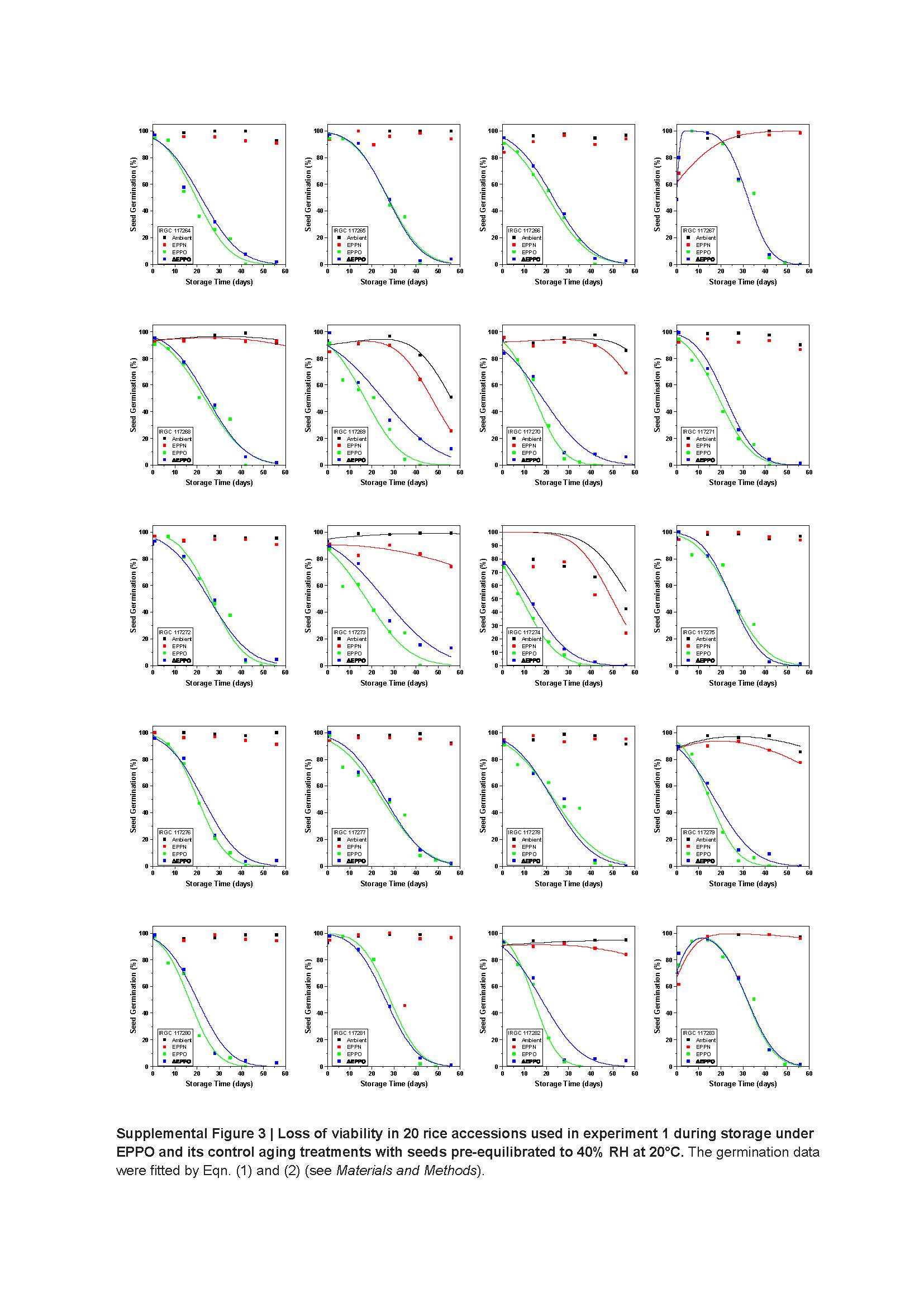

Supplement: Supplementary file 5 [file Image_3.jpeg]

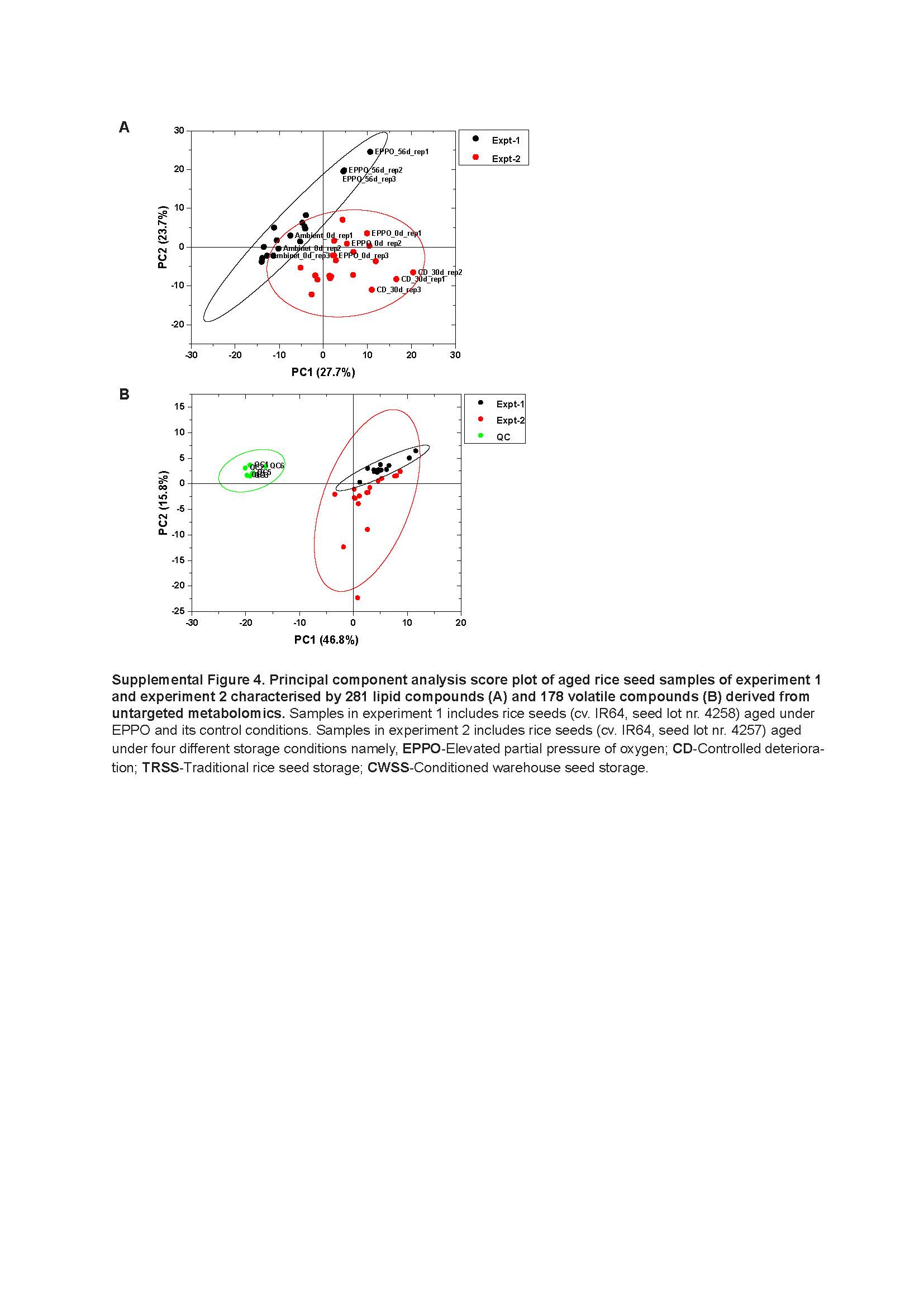

Supplement: Supplementary file 6 [file Image_4.jpeg]

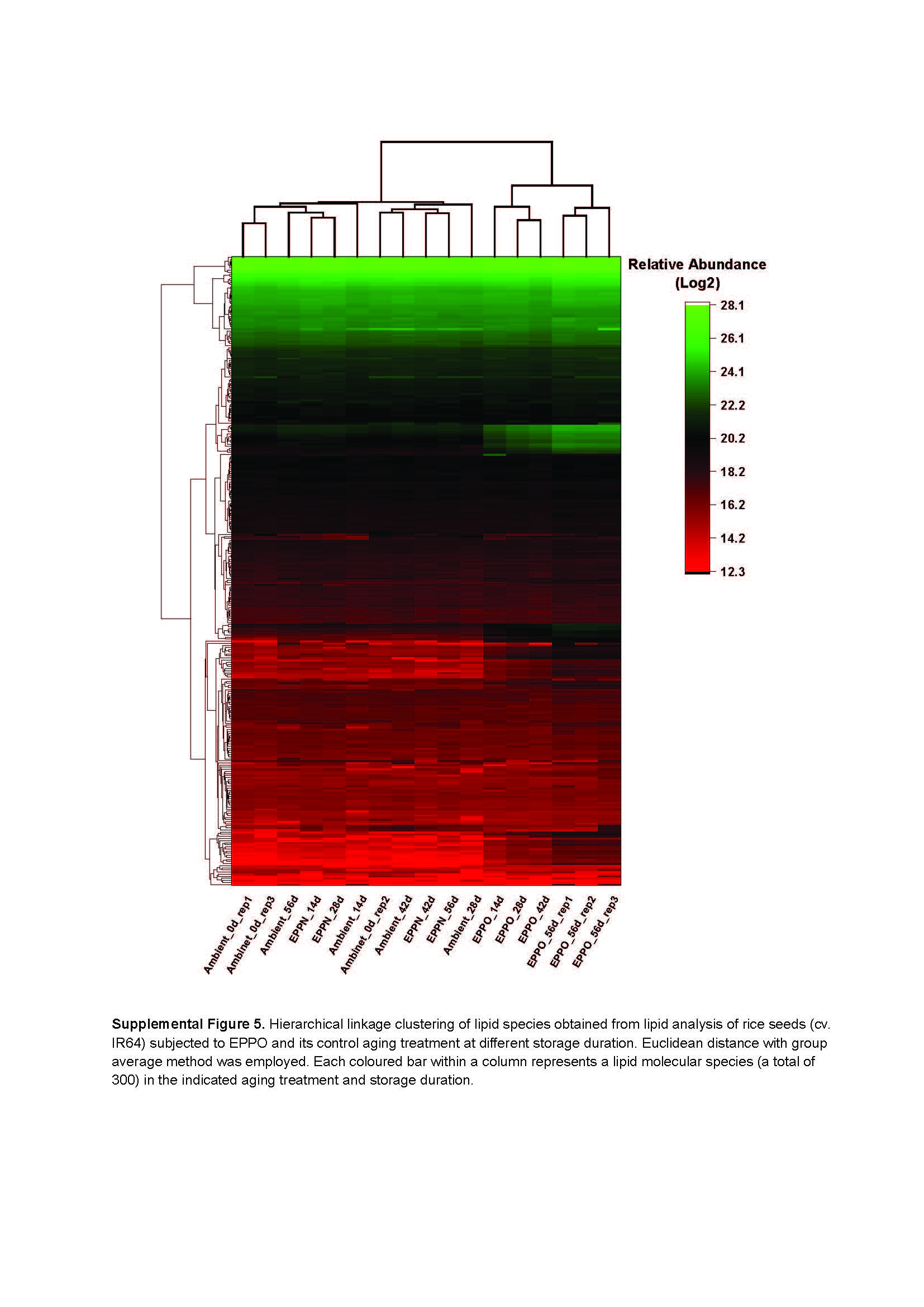

Supplement: Supplementary file 7 [file Image_5.jpeg]

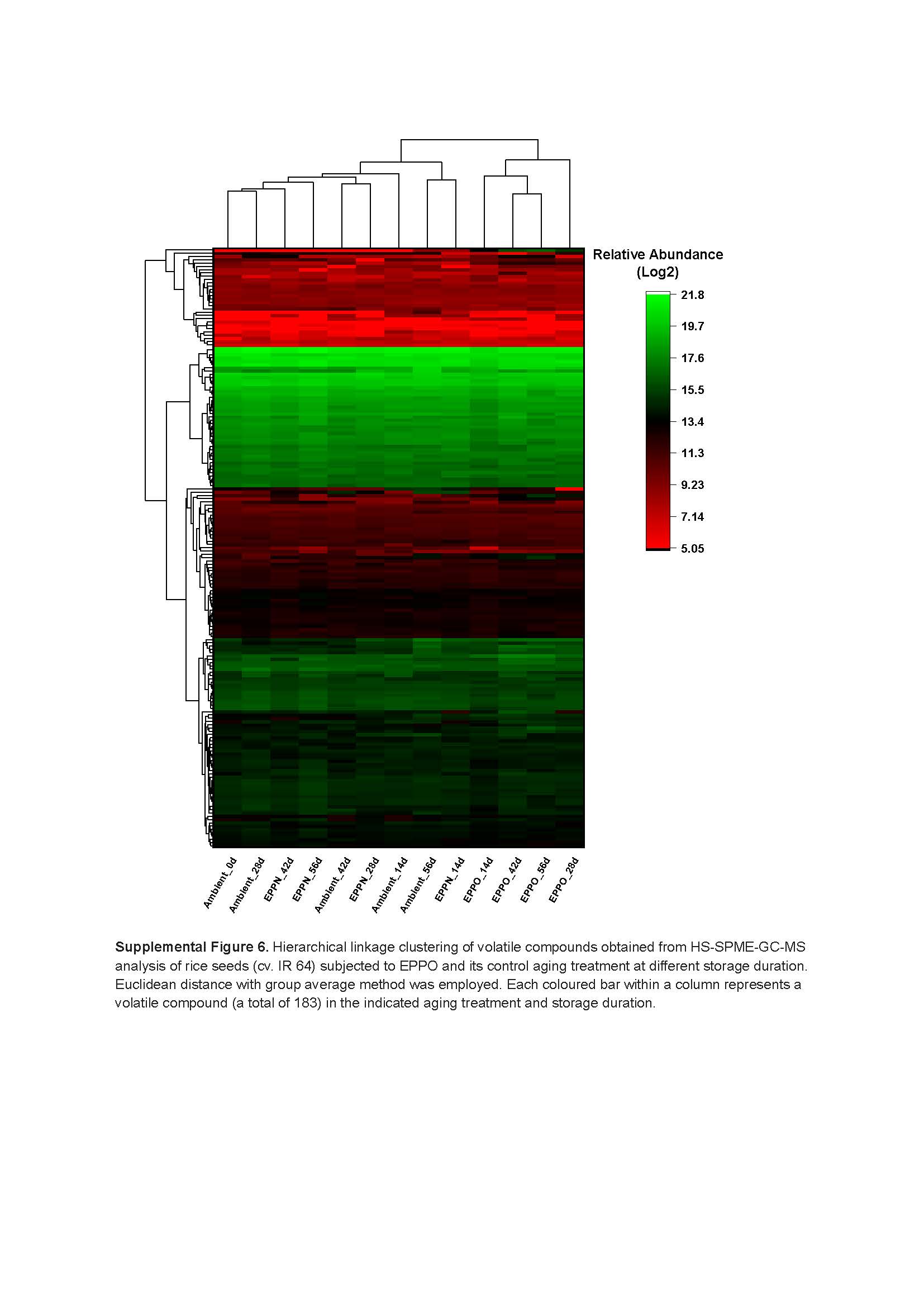

Supplement: Supplementary file 8 [file Image_6.jpeg]

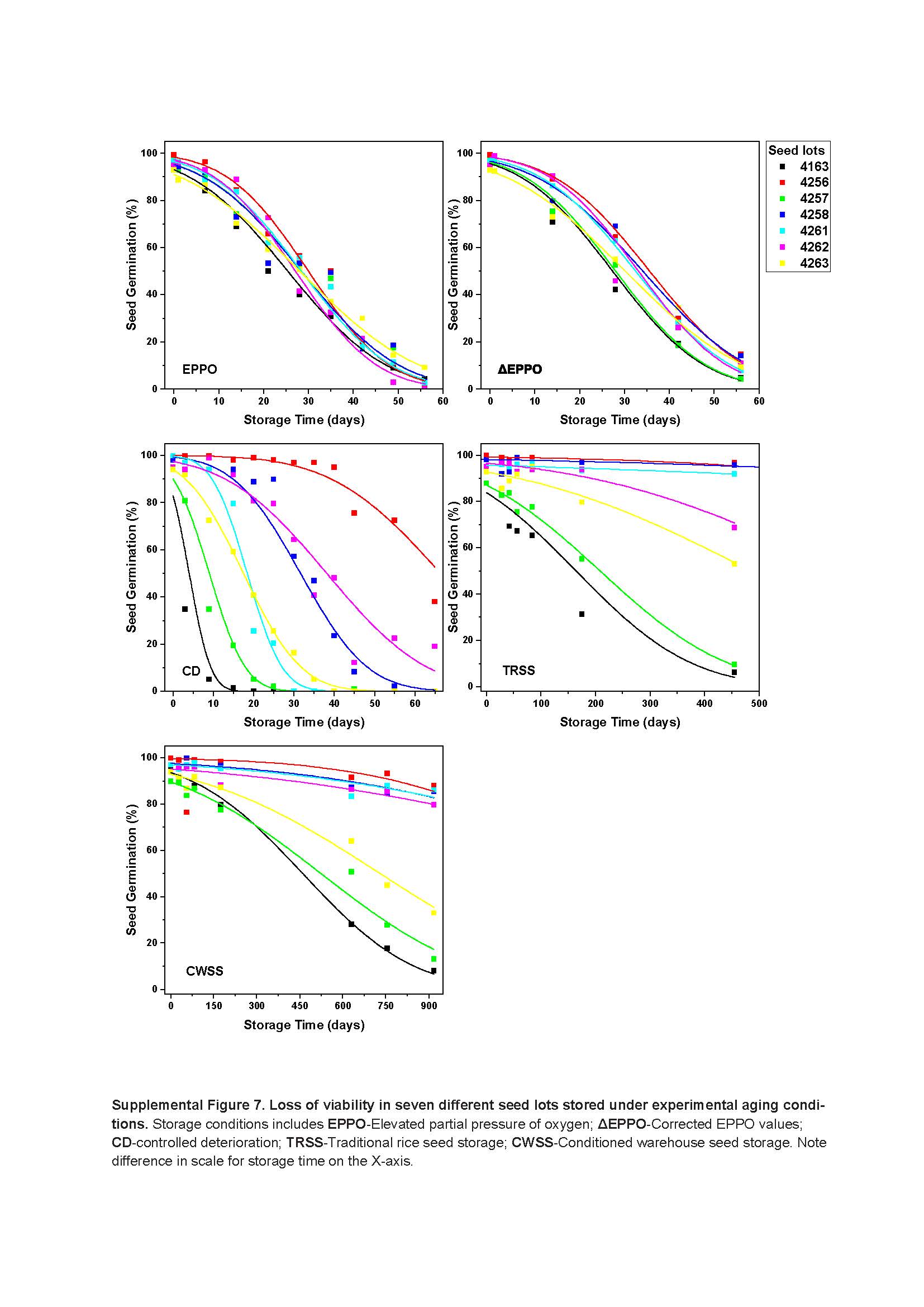

Supplement: Supplementary file 9 [file Image_7.jpeg]

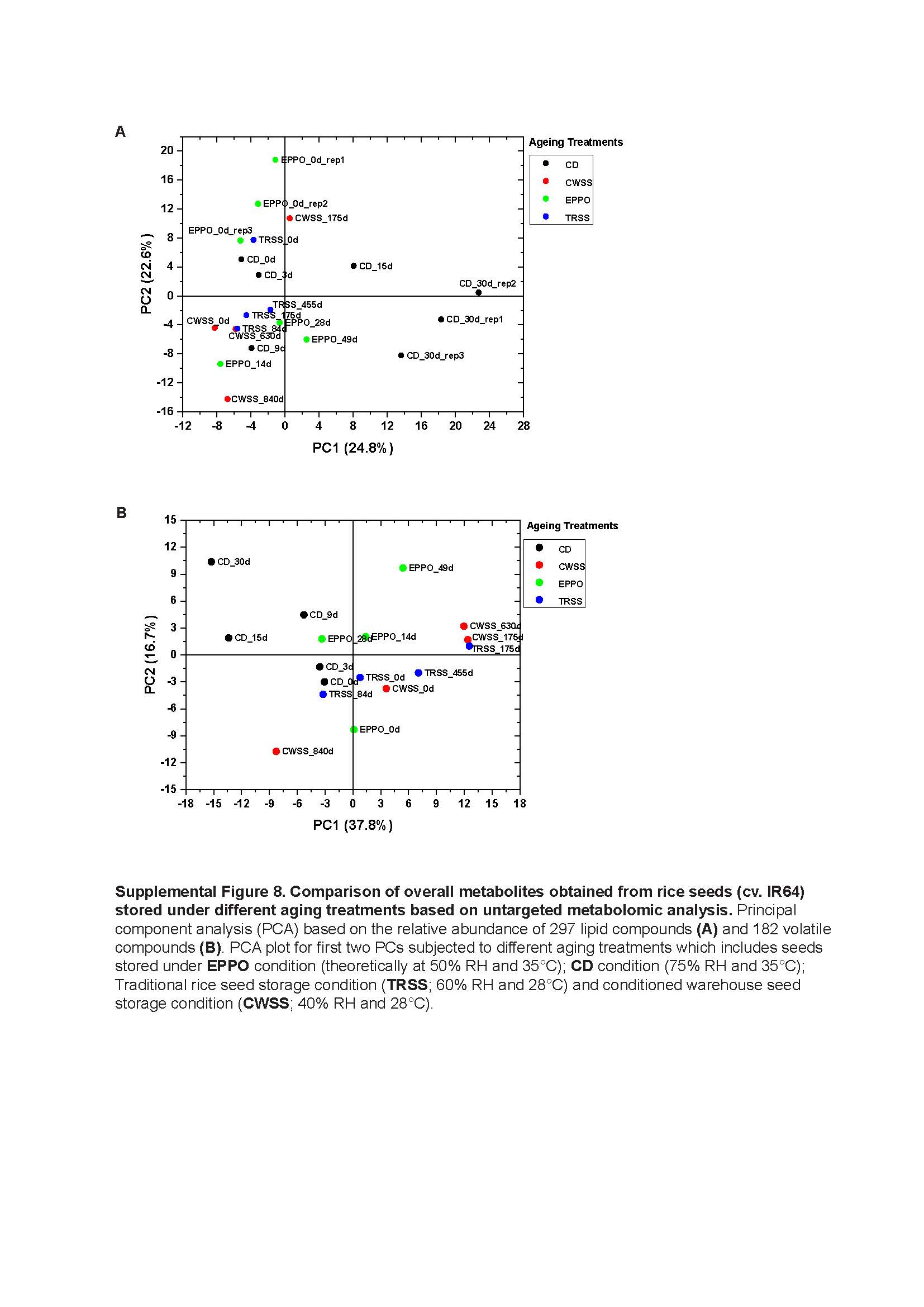

Supplement: Supplementary file 10 [file Image_8.jpeg]
