# Supplementary material for: Experimental rice seed aging under elevated oxygen pressure: Methodology and mechanism
Source: Front Plant Sci. 2022 Dec 1;13:1050411. doi: 10.3389/fpls.2022.1050411 (PMC9751813; doi:10.3389/fpls.2022.1050411)
Supplement: Supplementary file 12 [file Table_2.docx]

**Supplemental Table 2. Gas concentrations in different aging treatments (1 MPa = 10 bar).**

| **Storage Container** | **Gas used to fill the container** | **Ageing Treatment** | **Partial Pressure (in MPa)** | | | **Total Pressure**  **(in MPa)** |
| --- | --- | --- | --- | --- | --- | --- |
|  |  |  | **Nitrogen (**P_N2_**)** | **Oxygen (**P_O2_**)** | **Other Gasses (**P_Oth_**)** |  |
| Kilner Jar | Air | Ambient | 0.078 | 0.021 | 0.001 | 0.1 |
| Steel Tank | Nitrogen | Elevated Partial pressure of Nitrogen (EPPN) | 19.9 | 0.021 | 0.001 | 20.0 |
| Steel Tank | Air | Elevated Partial Pressure of Oxygen (EPPO) | 15.6 | 4.2 | 0.2 | 20.0 |
