# Supplementary material for: Experimental rice seed aging under elevated oxygen pressure: Methodology and mechanism
Source: Front Plant Sci. 2022 Dec 1;13:1050411. doi: 10.3389/fpls.2022.1050411 (PMC9751813; doi:10.3389/fpls.2022.1050411)
Supplement: Supplementary file 14 [file Table_4.docx]

**Supplemental Table 4.** **Seed longevity parameters derived from probit analysis of total germination data upon removal from storage for seven rice seed samples under dry-EPPO aging conditions in experiment 2.**

| **Seed Lot Nr.** | **Aging Condition** | **K*_i_* (s.e.)** | **σ^–1^ (s.e.)** | ***P_50_* (in d)** |
| --- | --- | --- | --- | --- |
| 4163 | EPPO | 1.40 (0.09) | 0.057 (0.003) | 24.7 |
|  | ΔEPPO | 1.53 (0.13) | 0.059 (0.004) | 26.01 |
|  | CD | 0.89 (0.13) | 0.292 (0.031) | 3 |
|  | TRSS | 0.98 (0.08) | 0.006 (0.001) | 151.7 |
|  | CWSS | 1.32 (0.08) | 0.003 (0.000) | 442 |
| 4256 | EPPO | 1.99 (0.11) | 0.066 (0.004) | 30 |
|  | ΔEPPO | 2.00 (0.14) | 0.057 (0.004) | 35.01 |
|  | CD | 3.56 (0.22) | 0.058 (0.005) | 61.9 |
|  | TRSS | 2.22 (0.33) | 0.001 (0.001) | 2528.9 |
|  | CWSS | 2.46 (0.22) | 0.001 (0.000) | 1715.2 |
| 4257 | EPPO | 1.60 (0.09) | 0.056 (0.003) | 28.7 |
|  | ΔEPPO | 1.73 (0.12) | 0.062 (0.004) | 27.71 |
|  | CD | 1.25 (0.11) | 0.147 (0.010) | 8.5 |
|  | TRSS | 1.12 (0.07) | 0.005 (0.000) | 209.3 |
|  | CWSS | 1.26 (0.08) | 0.002 (0.000) | 526.3 |
| 4258 | EPPO | 1.56 (0.11) | 0.055 (0.003) | 28.5 |
|  | ΔEPPO | 1.76 (0.14) | 0.052 (0.004) | 33.78 |
|  | CD | 2.71 (0.15) | 0.083 (0.004) | 32.6 |
|  | TRSS | 2.07 (0.21) | 0.001 (0.001) | 1479.6 |
|  | CWSS | 2.13 (0.19) | 0.001 (0.000) | 1598.8 |
| 4261 | EPPO | 1.77 (0.1) | 0.061 (0.003) | 28.9 |
|  | ΔEPPO | 2.01 (0.16) | 0.061 (0.004) | 32.82 |
|  | CD | 2.94 (0.21) | 0.162 (0.011) | 18.1 |
|  | TRSS | 1.80 (0.22) | 0.001 (0.001) | 1925 |
|  | CWSS | 1.86 (0.18) | 0.001 (0.000) | 1856.5 |
| 4262 | EPPO | 2.08 (0.15) | 0.075 (0.005) | 27.8 |
|  | ΔEPPO | 1.93 (0.15) | 0.061 (0.004) | 31.62 |
|  | CD | 1.95 (0.12) | 0.052 (0.003) | 37.6 |
|  | TRSS | 1.82 (0.14) | 0.003 (0.000) | 630.1 |
|  | CWSS | 1.58 (0.17) | 0.001 (0.000) | 2010.8 |
| 4263 | EPPO | 1.31 (0.1) | 0.047 (0.003) | 27.9 |
|  | ΔEPPO | 1.41 (0.1) | 0.047 (0.003) | 30.24 |
|  | CD | 1.56 (0.1) | 0.090 (0.005) | 17.3 |
|  | TRSS | 1.76 (0.2) | 0.004 (0.001) | 467.4 |
|  | CWSS | 1.46 (0.09) | 0.002 (0.000) | 724.9 |

**EPPO**-Elevated partial pressure of oxygen; **ΔEPPO**-corrected EPPO values; **CD**-controlled deterioration; **TRSS-**traditional rice seed storage; **CSWS-**conditioned warehouse seed storage; **K*i***- initial viability in NED; **σ^–1^-**length of time for viability to fall by 1 NED**; *P*_50_-**length of time for viability to fall to 50%**; d**-days; **s.e.**- standard error.
